# Generation of a pancreas derived hydrogel for the culture of hiPSC derived pancreatic endocrine cells

**DOI:** 10.1038/s41598-024-67327-9

**Published:** 2024-09-04

**Authors:** Constantin Berger, Markus Glaser, Anna-Lena Ziegler, Valentina Neukel, Fabiola Walz, Daniela Zdzieblo

**Affiliations:** 1 Chair Tissue Engineering and Regenerative Medicine, University Hospital Würzburg, Würzburg, Germany; 2 Project Center for Stem Cell Process Engineering, Fraunhofer Institute for Silicate Research, Würzburg, Germany; 3 Translational Center Regenerative Therapies, Fraunhofer Institute for Silicate Research, Würzburg, Germany

**Keywords:** Induced pluripotent stem cells, Stem-cell differentiation, Tissue engineering, Biomaterials, Biomaterials - cells

## Abstract

Stem cell-derived β-cells (SC-BCs) represent a potential source for curing diabetes. To date, in vitro generated SC-BCs display an immature phenotype and lack important features in comparison to their bona-fide counterparts. Transplantation into a living animal promotes SC-BCs maturation, indicating that components of the in vivo microenvironment trigger final SC-BCs development. Here, we investigated whether cues of the pancreas specific extracellular matrix (ECM) can improve the differentiation of human induced pluripotent stem cells (hiPSCs) towards β-cells in vitro. To this aim, a pancreas specific ECM (PanMa) hydrogel was generated from decellularized porcine pancreas and its effect on the differentiation of hiPSC-derived pancreatic hormone expressing cells (HECs) was tested. The hydrogel solidified upon neutralization at 37 °C with gelation kinetics similar to Matrigel. Cytocompatibility of the PanMa hydrogel was demonstrated for a culture duration of 21 days. Encapsulation and culture of HECs in the PanMa hydrogel over 7 days resulted in a stable gene and protein expression of most β-cell markers, but did not improve β-cell identity. In conclusion, the study describes the production of a PanMa hydrogel, which provides the basis for the development of ECM hydrogels that are more adapted to the demands of SC-BCs.

## Introduction

In the last years, several protocols for the directed differentiation of stem cell-derived β-cells (SC-BCs) have been published, demonstrating increasing efficiencies and improved β-cell signatures^1–7^. Nevertheless, in vitro generated SC-BCs predominantly display a fetal phenotype lacking important transcriptional, metabolic and functional features compared to β-cells from human adult islets^4,8^. SC-BC immaturity can be partially overcome by transplantation into a living animal^9^. Upon maturation, SC-BCs are able to ameliorate diabetes in mice^3,10,11^ and non-human primates^7^, suggesting that the key to β-cell maturation might lie in the in vivo microenvironment.

One essential factor of the in vivo microenvironment is the extracellular matrix (ECM). The ECM displays a non-cellular meshwork composed of secreted proteins and polysaccharides. In addition to providing structural support, the ECM acts as a signaling hub regulating important cellular functions such as cell survival^12^, proliferation^13^ and differentiation^14^. Studies on isolated human islets link the disruption of the islet ECM to an increased cell death of endocrine cells^15^. Apart from this, effects of the ECM on islet survival^12,16^, Insulin expression^17,18^, β-cell polarization^19^ and function^20^ have been reported.

Given the elementary role of the native ECM in islet physiology, the implementation of the ECM presents a promising approach to promote the performance of β-cells in vitro. ECM-derived hydrogels provide a useful strategy to accomplish this task, as they combine the advantage of the natural ECM composition with the benefits of a hydrogel (improved standardization, 3D culture, modifiable). Such hydrogels are usually generated by solubilization of solid ECM structures, followed by reassembly of ECM proteins into a water-swollen network^21^. Tissue specific ECM hydrogels have been generated from different tissues, including small intestine^22,23^, urinary bladder^24^, heart^25^, skin^26^, brain^27^, kidney^28^, liver^29^, lung^30^ and pancreas^31,32^. The tissue origin of the ECM plays a crucial role in terms of ECM composition, physico-structural characteristics and cell signaling^33^. Accordingly, it could be shown that ECM hydrogels derived from gastrointestinal tissue support the maintenance of small intestinal organoids^23,34^. Tremmel et al. further demonstrated a beneficial effect of a pancreas specific hydrogel on the survival and function of mature primary human islets^32^. This raises the question of whether a pancreas ECM also improves the terminal development of immature human induced pluripotent stem cell (hiPSC)-derived endocrine cells.

To tackle this question, we generated a pancreas specific ECM (PanMa) hydrogel from decellularized porcine pancreas, which we recently introduced^33^, and investigated its effect on the differentiation and β-cell identity of hiPSC-derived pancreatic hormone expressing cells (HECs). The produced PanMa hydrogel shows a pH and temperature sensitive gelation with gelation kinetics similar to Matrigel. We further show that the PanMa hydrogel is applicable in cell culture and maintains a stable expression of β-cell markers, but does not improve β-cell identity. Thus, the generated PanMa hydrogel provides a basis for further adaptation to the demands of β-cells.

## Results

### Reduced exposure time to the decellularization agent does not improve preservation of the laminin network in the PanMa

**Figure 1:**
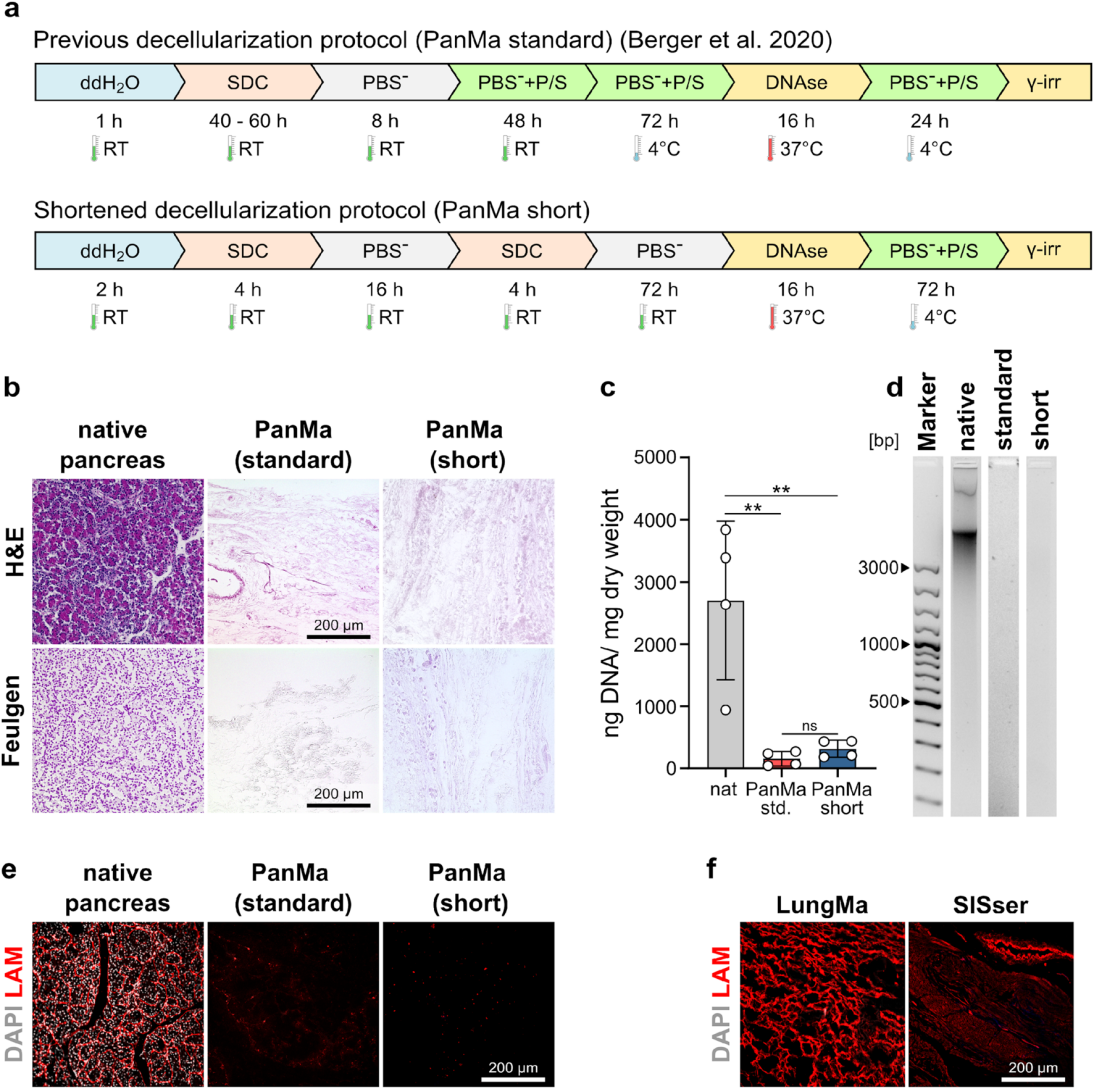
A shortened exposure time to sodium deoxycholate does not prevent laminin loss. (**a**) Illustration of the decellularization process of a standard protocol (upper scheme) and a protocol with reduced exposure time of the tissue to sodium deoxycholate (lower scheme). (**b**) Representative images of Haematoxylin and Eosin (H&E) and Feulgen staining of the native pancreas and PanMa scaffolds generated according to the standard or short decellularization protocol (n = 4). (**c**) DNA content of the native tissue in comparison to the produced PanMa scaffolds. Data represent mean ± SD (n = 4). ns (not significant), P > 0.05, **P < 0.01, one-way ANOVA with Tukey’s multiple comparisons test. Detailed information about the statistical testing, the respective replicate numbers and exact p-values is given in supplementary Table 1. (**d**) Qualitative DNA assessment of DNA isolated from native pancreas tissue and the generated PanMa scaffolds using electrophoresis (n = 4). The presented images present only parts of the original gels. The uncropped images can be found in supplementary Fig. 4. (**e**) Representative immunofluorescent stainings of native pancreas tissue and the PanMa scaffolds produced by the respective decellularization protocols labelled with antibodies against laminin (red) and counterstained with DAPI (grey) (n = 2). (**f**) Immunofluorescent images of acellular scaffolds produced by decellularization of lung tissue (LungMa) or intestinal tissue (SISser). Sections are stained for laminin (red) and counterstained with DAPI (n = 2). Abbreviations: DNAse: DNAse I digest, PBS^−^: phosphate buffered solution without calcium and magnesium, P/S: penicillin/streptomycin, SDC: sodium deoxycholate, γ-irr: γ-irradiation.

The generation of a tissue specific ECM hydrogel requires a suitable material source that closely mimics the ECM composition of the respective tissue. For the generation of a pancreas specific hydrogel, we therefore used the recently introduced pancreas specific extracellular matrix scaffold (PanMa)^33^. In this study, we observed a destruction of the laminin network in the PanMa scaffold, while laminin fragments were still detected in mass spectrometry^33^. In an attempt to improve the preservation of the laminin structure, we therefore tested a shortened decellularization protocol (PanMa short) and compared its outcome to our previously established protocol for PanMa generation (PanMa standard) (Fig. 1a).

In the shortened protocol, the cellular detergent sodium deoxycholate was applied in two perfusion steps (4 h each) with an intermediate PBS^-^ washing step. This allowed a reduction of the exposure time of the tissue to the decellularization reagent from 40–60 h to 8 h compared to the standard protocol. The overall tissue retention was comparable between both protocols, as indicated by H&E staining (Fig. 1b). Feulgen staining showed a complete removal of DNA from scaffolds generated with the standard protocol, while few DNA remnants were detected in scaffolds produced with the shortened protocol (Fig. 1b), indicating incomplete DNA removal. Accordingly, quantification of the residual DNA revealed a slightly higher DNA content in scaffolds generated with the shortened protocol (316.7 ± 138.2 ng DNA/mg dry weight) compared to those produced with the standard protocol (157.5 ± 115.9 ng DNA/mg dry weight), but this was not statistically significant (Fig. 1c). Qualitative assessment of the residual DNA showed no DNA fragments larger than 200 base pairs (Fig. 1d), which is suggested as a cut-off size for DNA to avoid adverse effects^35^. In contrast to the initial hypothesis, reduced exposure times of the tissue to the decellularization agent did not prevent the loss of laminin (Fig. 1e). Instead, no intact laminin network could be detected in scaffolds produced with the standard or shortened protocol. Laminin networks could be preserved in scaffolds which were derived from lung (LungMa) and intestinal tissue (SISser) by sodium deoxycholate-based decellularization (Fig. 1f), demonstrating that the decellularization agent is not the leading cause for the destruction of the laminin network.

To figure out the cause for laminin network disruption, we analyzed samples at different steps during the shortened decellularization process (Fig. S1a). Interestingly, the laminin network revealed signs of degradation already after perfusion with ddH_2_O (Fig. S1b), which was not the case for lung and intestine decellularization (Figs. S2b and S3b). Subsequent perfusion with sodium deoxycholate led to a removal of laminin structures in the pancreatic lobes but did not affect the laminin structures of vessels (Fig. S1b). Notably, γ-irradiation of the PanMa resulted in a destruction of residual laminin within vessel structures. Surprisingly, DNA levels were diminished after perfusion of the pancreas with ddH_2_O as shown by Feulgen staining and DNA quantification (Fig. S1c,d). This was not observed during the production of LungMa and SISser (Figs. S2c,d and S3c,d).

Altogether, our data show that laminin network destruction during decellularization is due to a pancreas specific effect and independent of sodium deoxycholate. Reduced exposure time to sodium deoxycholate did not lead to an improved decellularization process.

### Production of a pancreas specific hydrogel

**Figure 2:**
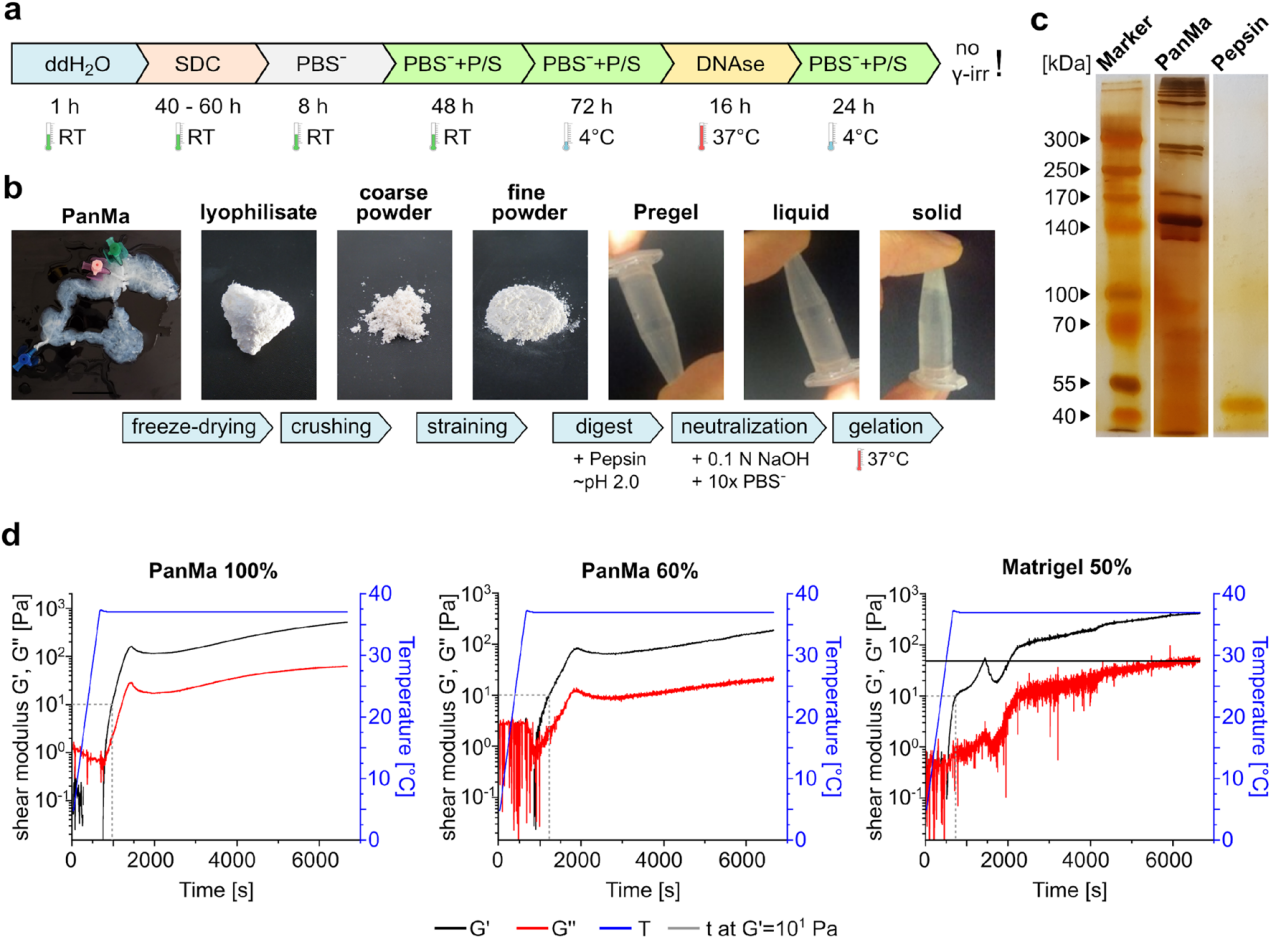
Production of a pancreas ECM hydrogel. (**a**) Graphic depiction of the decellularization protocol used for generating PanMas for the subsequent hydrogel production. (**b**) Images showing the process of PanMa hydrogel generation. The arrows below describe the individual working steps. (**c**) Silver staining of the Pepsin digested PanMa to visualize the protein fragments contained in the PanMa digest. Pepsin solution was used as a negative control. Uncropped images are shown in supplementary Fig. 4. (n = 2). (**d**) Gelation dynamics of the undiluted (100%) and diluted (60%) PanMa hydrogel in comparison to diluted (50%) Matrigel. The graphs show the storage (Gʹ, black line) and loss (G″, red line) modulus of the hydrogels during gelation initiated by temperature increase (blue line) (n = 2).

Since the shortened protocol did not result in an improved PanMa, we used the standard protocol to generate pancreatic scaffolds for the ECM hydrogel production. We further found that γ-irradiation of the solid PanMa scaffolds prevented a subsequent gel formation, indicating that γ-irradiation abolishes the crosslinking ability of contained proteins. Therefore, γ-irradiation was omitted in the production of PanMa scaffolds used for hydrogel production (Fig. 2a). To convert the solid PanMa into a hydrogel, the PanMa was lyophilized, crushed into a powder and subsequently digested with pepsin. Neutralization of salt concentration and pH resulted in gelation of the pregel at 37 °C (Fig. 2b).

Analyses of the pregel protein content using silver staining revealed the presence of proteins of different size (Fig. 2c). Next to smaller proteins and protein fragments (≤ 70 kDa), medium sized (approx. 150 kDa) and large proteins (≥ 300 kDa) were detected in the PanMa hydrogel, demonstrating the preservation of high molecular weight ECM proteins. Using rheological measurements, we investigated the gelling behavior of the PanMa hydrogel, which is a substantial characteristic of hydrogels. The PanMa hydrogel was measured undiluted (100%) as the maximum concentration, and at the highest dilution forming a stable hydrogel (60%). For comparison, we used Matrigel, the gold standard for many hydrogel-based cell culture applications, in a 1:1 diluted concentration to simulate cell culture conditions. Measuring the storage (Gʹ) and loss (G″) modulus during gelation of 50% Matrigel at 37 °C revealed five different phases during Matrigel solidification (Fig. 2d). Upon heating to 37 °C, Gʹ showed a steep increase (phase 1) and a subsequent second solidification phase with a flattened slope and a short rise (phase 2). Next, a drop of Gʹ occurred (phase 3), followed by another increase of Gʹ (phase 4) and the transition into a plateau phase (phase 5). In contrast, 100% and 60% PanMa hydrogel solidified in three phases, comparable to phase 1, 3 and 5 of the Matrigel gelation process. Interestingly, gelation of the PanMa hydrogel was induced after reaching a temperature of 37 °C and thus was delayed compared to 50% Matrigel. Accordingly, the initial gelation time, here defined as the time required to reach a storage modulus of 10 Pa, was longer for the 100% PanMa (968 s) in comparison to 50% Matrigel (748 s). Dilution of the PanMa hydrogel to 60% delayed the gelation process by roughly 276 s (1,244 s). Storage and loss modulus of the solidified hydrogels after 100 min were comparable between 100% PanMa (Gʹ = 400 Pa, G″ = 65 Pa) and 50% Matrigel (Gʹ = 420 Pa, G″ = 50), indicating similar viscoelastic properties of both hydrogels. The 60% PanMa exhibited decreased storage and loss moduli (Gʹ = 200 Pa, G″ = 20 Pa), demonstrating a loss of rigidity due to dilution.

Summarized, these findings confirm the ability of the PanMa hydrogel to form a solid hydrogel with rheological characteristics similar to those of Matrigel.

### The PanMa hydrogel is cytocompatible and suitable for the encapsulation of pancreatic endocrine spheroids

**Figure 3:**
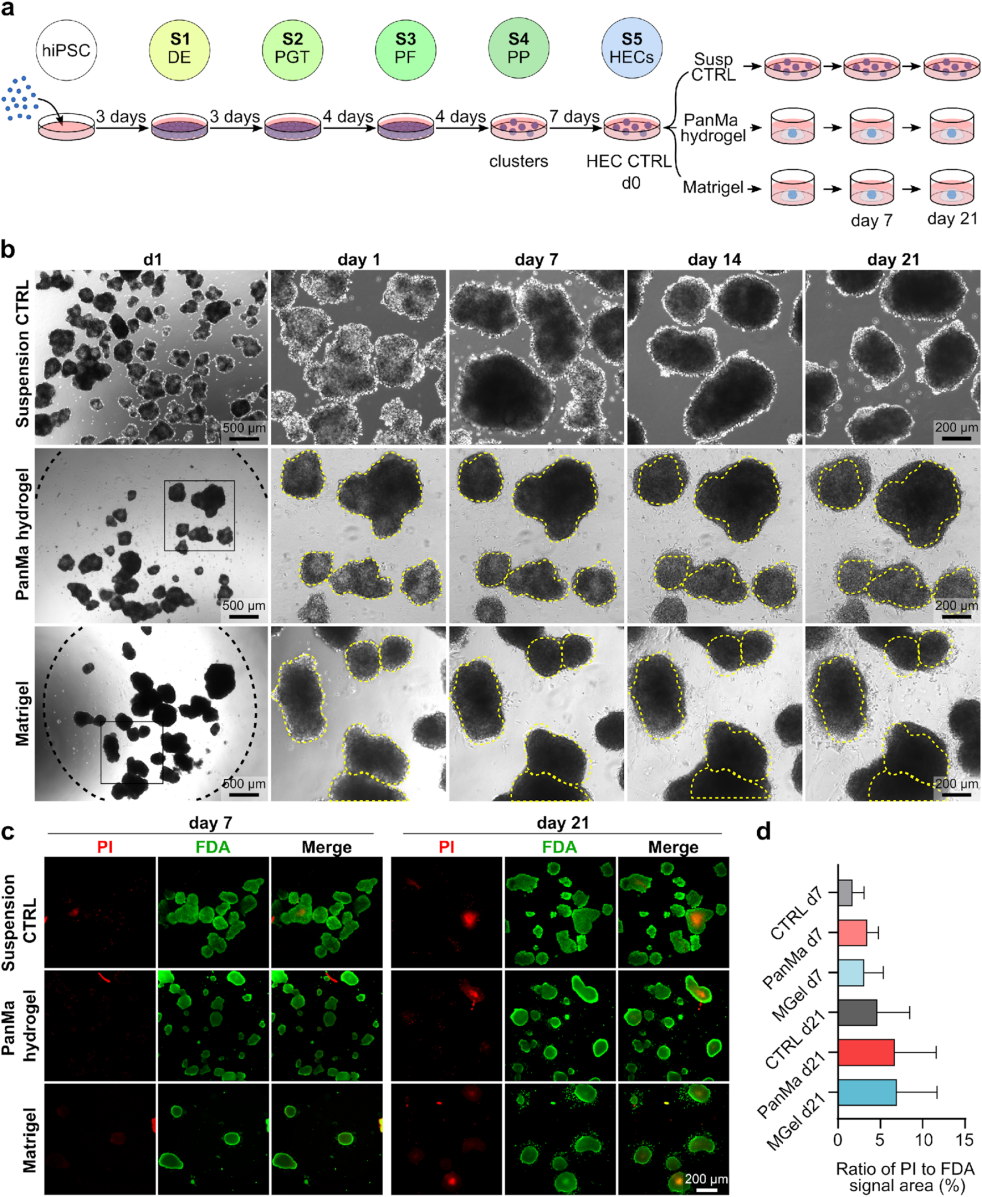
The PanMa hydrogel allows encapsulation and culture of hiPSC-derived HECs. (**a**) Graphical illustration of the experiment. hiPSCs were differentiated towards HECs, encapsulated in PanMa hydrogel or Matrigel and cultured for up to 21 days. HECs cultured in suspension were used as control (CTRL). (**b**) Brightfield images of HECs in suspension, encapsulated in PanMa hydrogel and Matrigel at day 1, 7, 14 and 21. Dotted lines mark the border of the hydrogel drop (black dotted line) and spheroid shape at day 1 (yellow dotted line) (n = 3). (**c**) Microscopic images of HECs stained with FDA/PI to label living (FDA^+^) and dead (PI^+^) cells (n = 3). (**d**) Quantified fluorescent signal area for FDA and PI. Shown is the ratio of PI^+^ area to FDA^+^ area per condition. Data represent mean ± SD (5–12 pictures of n = 3 biological replicates). One-way ANOVA with Sidak’s multiple comparisons test. Detailed information about the statistical testing, the respective replicate numbers and exact p-values is given in supplementary Table 1.

To test whether the PanMa hydrogel can improve the β-cell identity of developing endocrine cells, we differentiated hiPSCs towards pancreatic endocrine cells using the suspension protocol by Rezania et al.^2,3^ and encapsulated the generated HECs in PanMa hydrogel. For encapsulation, a 75% PanMa hydrogel diluted with medium was used, which proved to be a compromise between a reduction of material and rapid gelation (Fig. 3a). As the PanMa lacked a comprehensive laminin network, we used Matrigel, a laminin-containing hydrogel^36^, for comparison. HECs cultured in suspension were used as a control.

The cell-laden PanMa hydrogel solidified at 37 °C within 15 min resulting in hydrogel droplets (black dotted line) containing the single spheroids (yellow dotted line) (Fig. 3b). Prolonged culture of the encapsulated HECs resulted in a morphological change of the HECs towards more circular spheroids, indicating that the stable PanMa hydrogel is flexible enough to allow re-shaping of biological structures. A similar re-structuring was observed in spheroids cultured in suspension and in Matrigel. In contrast to the suspension culture, HECs encapsulated in the PanMa hydrogel appeared less demarcated and dense from day 14 on. The same was observed in Matrigel, indicating that hydrogel encapsulation led to a decreased cellular cohesion. To exclude that this was due to a cytotoxic effect of the hydrogel, we examined cell viability using fluorescein diacetate (FDA, viable)/ propidium iodide (PI, dead) staining (Fig. 3c). No decreased cell viability or increased cell death could be observed in HECs encapsulated in PanMa hydrogel or Matrigel after 7 or 21 days in comparison to the suspension control, demonstrating the cytocompatibility of the PanMa hydrogel (Fig. 3d).

Summarized, the data prove the cytocompatibility of the PanMa hydrogel and suggest a stable culture of HEC spheroids in terms of shape for up to 14 days after encapsulation.

### Short term culture in the PanMa hydrogel results in a stable β-cell gene expression

Next, the effect of the PanMa hydrogel encapsulation on endocrine differentiation with a particular focus on β-cell development was investigated. To this end, gene expression analysis of genes accompanying β-cell differentiation was performed on HECs in suspension and on HECs encapsulated in the PanMa hydrogel for 7 or 21 days. We were particularly interested in the expression of the transcription factors *PDX1*, which represents an early marker of pancreatic lineage development that is sustained in developing and mature β-cells^37,38^, *NKX6.1*, which is required for β-cell lineage commitment^37,39^, as well as *MAFA*, a marker for advanced β-cell maturation^40,41^. The expression of all three factors is a hallmark of β-cell identity and indispensable for β-cell specification and maturation^39^. Moreover, we investigated the expression of the pancreatic hormones *INS*, *GCG* and *SST,* which are expressed in β-, α- and δ-cells, respectively.

**Figure 4:**
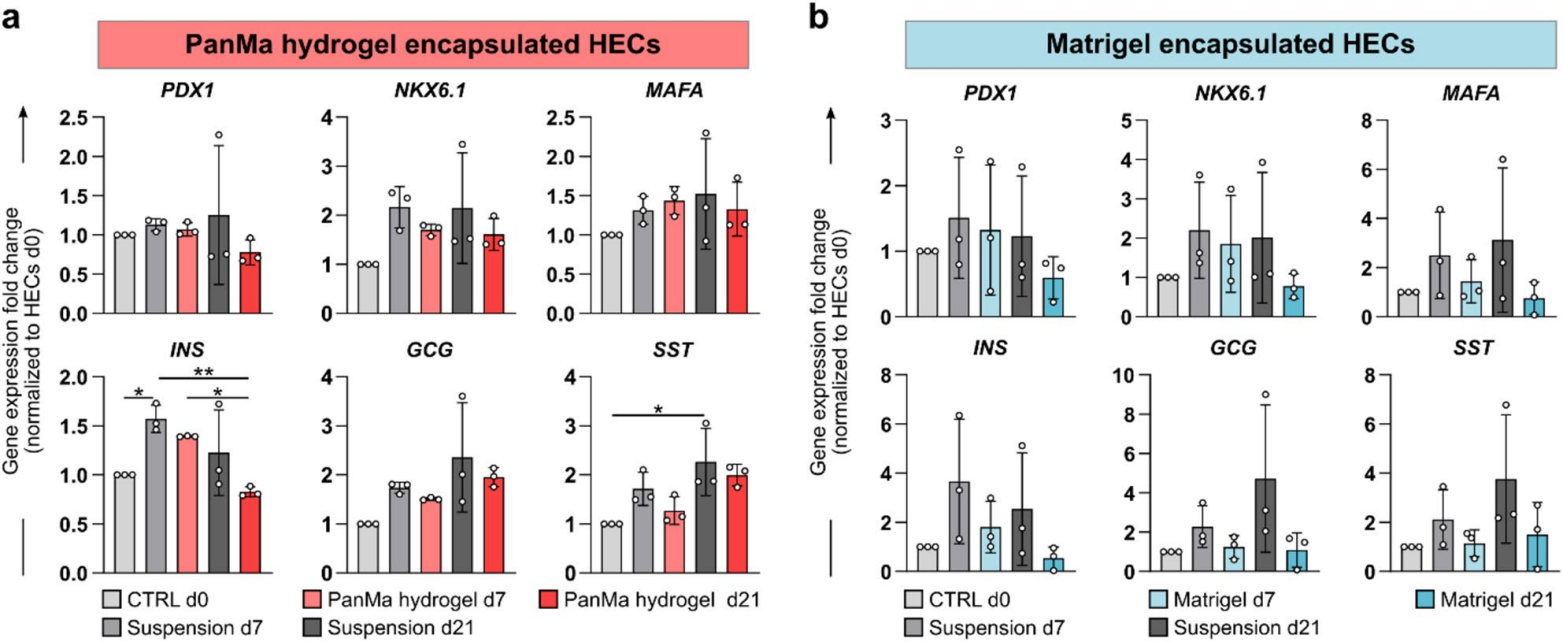
PanMa hydrogel encapsulation does not affect gene expression of endocrine genes in HECs. (**a**,**b**) Gene expression of HECs encapsulated in PanMa hydrogel (**a**) or Matrigel (**b**) at day 7 and 21 after encapsulation shown as fold change relative to expression in HECs (day 0) after differentiation from hiPSCs. HECs in suspension were used as control. Gene expression of Matrigel and PanMa hydrogel encapsulated HECs are shown separately as data were collected in independent experiments with individual control groups. Data are shown as mean ± SD (n = 3). *P < 0.05, **P < 0.01, one-way ANOVA with Tukey’s multiple comparisons test. Detailed information about the statistical testing, the respective replicate numbers and exact p-values is given in supplementary Table 1.

HECs in suspension showed a stable *PDX1* expression over the course of the experiment (Fig. 4a). *NKX6.1* and *MAFA* expression appeared to be slightly increased at day 7 and day 21 in suspension compared to CTRL day 0, indicating an ongoing differentiation with extended culture in differentiation medium. However, this was not statistically significant. Encapsulation of HECs in the PanMa hydrogel for seven days had no effect on *PDX1, NKX6.1* and *MAFA* gene expression compared to the time-matched suspension control. In case of the pancreatic hormones, a significant increase in *SST* and *INS* expression was observed in HECs in suspension over time. Encapsulation of HECs in the PanMa hydrogel had no significant impact on *GCG* and *SST* expression. In contrast, *INS* expression decreased from day 7 to day 21 after encapsulation, indicating a loss of β-cell phenotype with extended culture in the PanMa hydrogel. Similar to the PanMa hydrogel, no significant change was observed in the gene expression of *PDX1*, *NKX6.1* and *MAFA* in HECs encapsulated in Matrigel (Fig. 4b). A similar decrease in *INS* expression was observed in HECs at day 21 of Matrigel encapsulation, which however was not statistically significant.

### The PanMa hydrogel enables a continued differentiation of HECs

To gain a deeper insight into the cytoarchitecture of the encapsulated spheroids and the differentiation state of individual HECs, we employed immunolabeling of the β-cell markers PDX, NKX6.1 and MAFA as well as C-peptide (CPEP), GCG and SST to stain β-like, α-like and δ-like cells, respectively. Mature β-cells are supposed to be positive for PDX, NKX6.1, MAFA and CPEP and negative for GCG and SST.

**Figure 5:**
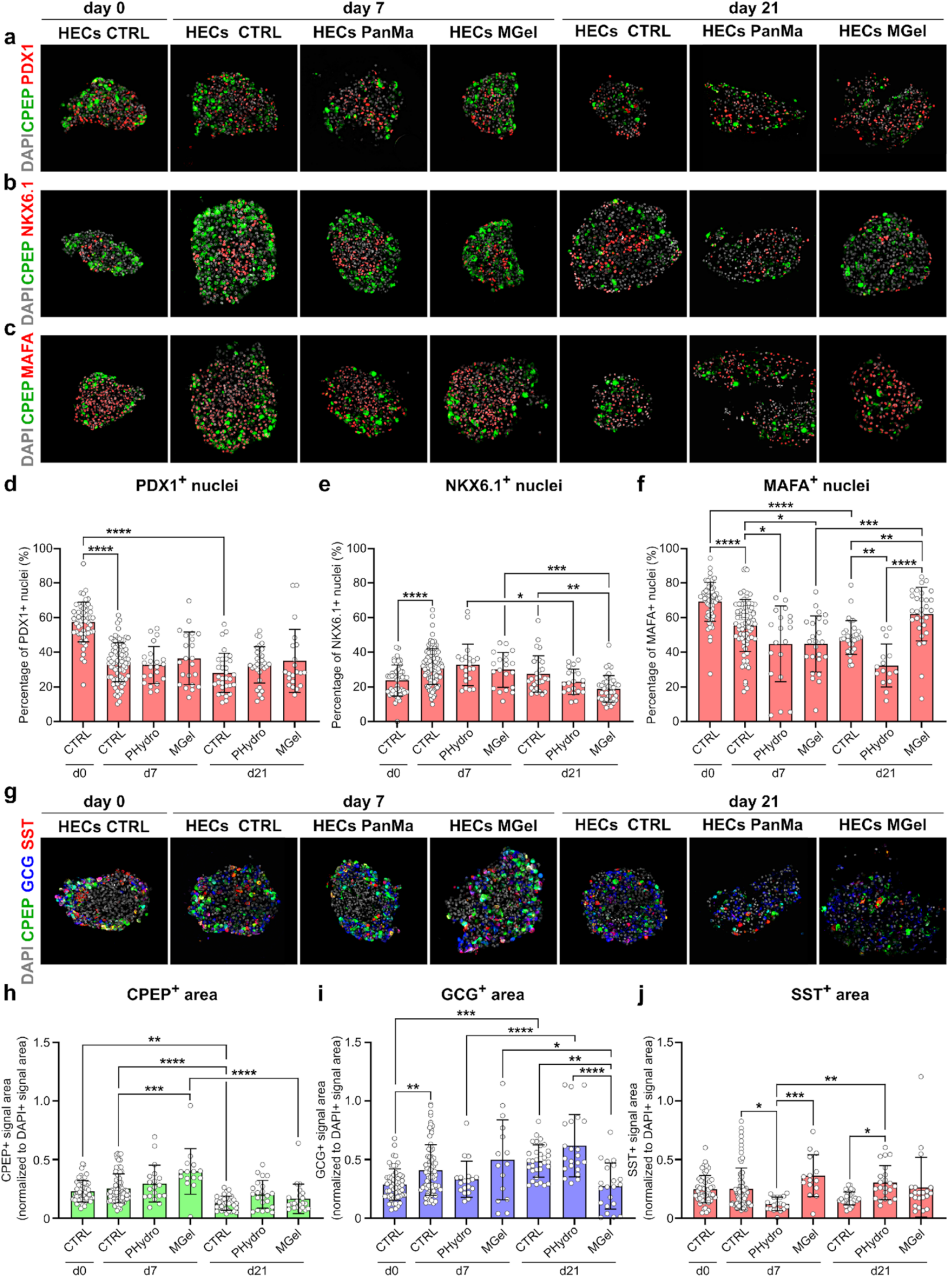
HECs maintain their endocrine phenotype for a limited time after hydrogel encapsulation. (**a**–**c**) Representative images of immunolabeled HECs encapsulated in PanMa hydrogel or Matrigel at day 7 and day 21 after encapsulation. HECs cultured in suspension are shown as control. Cells were stained for the transcription factors PDX1 (**a**, red), NKX6.1 (**b**, red), MAFA (**c**, red). All samples were counterstained with the β-cell marker C-Peptide (green) and DAPI for visualization of nuclei (grey). (**d**–**f**) Quantification of nuclei positive for PDX1 (**d**), NKX6.1 (**e**) or MAFA (**f**) displayed as the percentage of total nuclei (DAPI-labelled). Data are shown as mean ± SD (≥ 15 spheroids of n ≥ 3 biological replicates). Each data point represents an individual spheroid. *P < 0.05, **P < 0.01, ***P < 0.001, ****P < 0.0001, one-way ANOVA with Sidak’s multiple comparisons test. (**g**) Representative images of HECs after encapsulation or suspension stained for CPEP (green), GCG (blue) and SST (red) counterstained with DAPI (grey). (**h**–**j**) Quantification of CPEP (**h**), GCG (**i**) and SST (**j**) fluorescent signal area shown as ratio to total DAPI area. Data are shown as mean ± SD (≥ 15 spheroids of n ≥ 3 biological replicates). Each data point represents an individual spheroid. *P < 0.05, **P < 0.01, ***P < 0.001, ****P < 0.0001, one-way ANOVA with Sidak’s multiple comparisons test. Detailed information about the statistical testing, the respective replicate numbers and exact p-values is given in supplementary Table 1.

Cells positive for either PDX1, NKX6.1, MAFA or CPEP were found across all conditions (Fig. 5a–c). Image quantification revealed a decrease of PDX1^+^ cells with ongoing culture under control conditions from 58% ± 11% at day 0 to 34% ± 11% and 28% ± 11% at day 7 or day 21, respectively (Fig. 5d), suggesting that some HECs shut down PDX1-driven programs required for pancreatic lineage development. Encapsulation and culture in the PanMa hydrogel or Matrigel did not affect the number of PDX1^+^ cells. Under control conditions, the frequency of NKX6.1^+^ cells increased with ongoing culture from day 0 (24% ± 9%) to day 7 (32% + 10%) (Fig. 5e), indicating an enhanced β-cell commitment in this period. While HECs encapsulated in the PanMa showed no difference to the time-matched CTRLs, HECs in Matrigel exhibited a lower amount of NKX6.1^+^ cells at day 21 (19% ± 8%) compared to CTRL day 21 (27% ± 10%). The number of HECs positive for the β-cell maturation marker MAFA decreased with extended culture from day 0 (70% ± 11%) until day 7 (56% ± 15%) and day 21 (49% ± 10%) (Fig. 5f). Encapsulated spheroids exhibited a lower number of MAFA^+^ cells at day 7 in the PanMa hydrogel (45% ± 21%) and in Matrigel (45% ± 16%). Interestingly, at day 21, HECs encapsulated in Matrigel exhibited a larger number of MAFA^+^ cells (62% ± 15%) compared to the PanMa (33% ± 12%) and the time-matched CTRL (49% ± 10%).

Investigating the presence of CPEP, GCG and SST in HECs, we found that a large proportion of cells was positive for more than one hormone at day 0 of culture (Fig. 5g). With ongoing culture, HECs in all conditions appeared to contain more monohormonal cells, while GCG appeared to be the dominant hormone at day 21. Quantification of the fluorescent signal area for each hormone confirmed this impression. The CPEP^+^ area in the suspension CTRL appeared stable from day 0 (0.23 ± 0.09) until day 7 of culture (0.25 ± 0.12) (Fig. 5h). This was similar for HECs encapsulated in the PanMa hydrogel for 7 days (0.30 ± 0.15), while Matrigel encapsulation led to a slight increase in the proportion of CPEP^+^ cells (0.40 ± 0.19). Longer culture resulted in a decreased CPEP^+^ signal area in suspension (0.13 ± 0.06) as well as the PanMa hydrogel (0.21 ± 0.12, not significant) or Matrigel (0.17 ± 0.12), indicating a loss of β-cell identity over time. In contrast, the GCG^+^ area increased continuously from day 0 in suspension (0.29 ± 0.13) until day 21 (0.49 ± 0.14) (Fig. 5i), indicating a predisposition of the immature HECs for an α-cell fate. HECs encapsulated in the PanMa hydrogel showed no difference in the GCG^+^ area compared to the time-matched CTRLs. However, Matrigel-encapsulated spheroids exhibited a significantly smaller GCG^+^ area (0.27 ± 0.19) compared to the CTRL (0.49 ± 0.14), suggesting an effect of Matrigel on α-cell specification. For the δ-cell hormone SST, no change was observed in the suspension CTRL over time (0.25 ± 0.12, 0.25 ± 0.18 and 0.17 ± 0.05 in CTRL day 0, day 7 and day 21, respectively) (Fig. 5j). However, a higher SST^+^ signal area was found in HECs encapsulated in the PanMa hydrogel at day 21 (0.31 ± 0.14), indicating a promoted δ-cell differentiation in the PanMa hydrogel.

An important indication of β-cell identity is the presence of β-cell transcription factors within CPEP^+^ cells. According to the qualitative assessment, we found CPEP^+^/PDX1^+^ and CPEP^+^/MAFA^+^ cells at early and late time points across all conditions (Fig. 5a,c). CPEP^+^/PDX1^-^ and CPEP^+^/MAFA^-^ cells were rarely detected. Interestingly, CPEP^+^/NKX6.1^+^ cells were detected earliest at day 7, suggesting a continued differentiation with prolonged culture (Fig. 5b). Observing the appearance of CPEP^+^/NKX6.1^+^ cells in encapsulated HECs demonstrated that both the PanMa hydrogel and Matrigel enabled a continued differentiation. This finding is corroborated by the fact that after 7 days of culture, HECs in all conditions were mostly monohormonal, an important hallmark of maturing HECs.

Moreover, we observed a rearrangement of the spheroid cytoarchitecture in both suspension-cultured and encapsulated HECs. On day 0, CPEP^+^ cells were mostly detected in the outer regions and not in the center in a large number of spheroids. In contrast, day 7 spheroids exhibited an even distribution of CPEP^+^ cells in both outer regions and the center, indicating that rearrangement of the spheroid accompanies HEC differentiation. This was similarly observed in HECs encapsulated in PanMa hydrogel and Matrigel, demonstrating that encapsulation enables a spatial rearrangement of the spheroids.

Altogether, the data suggest that PanMa hydrogel and Matrigel enable a continued differentiation until day 7. While the PanMa had no effect on extended culture of HECs, Matrigel increased the frequency of MAFA^+^ cells and decreased GCG^+^ area, without affecting CPEP.

## Discussion

In the present study, we investigated the effect of a porcine pancreas ECM hydrogel on the differentiation of hiPSC-derived HECs. The presented hydrogel was produced from porcine pancreas decellularized with sodium deoxycholate. In an early phase of hydrogel production, we observed that γ-irradiation of the solid PanMa scaffold prevented the gelation of derived pregels. This suggests that γ-irradiation at doses > 25 kG alters peptide sequences required for crosslinking. Indeed, studies show that high doses of γ-irradiation lead to a decrease in scaffold elasticity and an increased susceptibility to proteolytic enzyme degradation^42^, suggesting that γ-irradiation initiates a structural reorganization of the ECM scaffold including destruction and formation of peptide crosslinks. In contrast to that, Giobbe et al. demonstrated gelation despite γ-irradiation of the ECM powder at a dosage of 17 kG for 10 h^23^, suggesting that dosages ≤ 17 kG present a workable compromise. Nevertheless, most protocols for the production of ECM hydrogels omit γ-irradiation and other sterilization steps. This emphasizes that the production process of the hydrogel, including lyophilization, HCl-solubilization and pepsin digest, is sufficient for a sterile hydrogel production and makes additional sterilization steps superfluous. Decellularization resulted in residual DNA contents higher than 50 ng/mg tissue, which is widely recognized as the threshold for ECM scaffolds intended for in vivo or in vitro use^35^. The high content might be explained by the fact that we employed whole organ decellularization, which might impede the removal of residual DNA. Analysis of DNA size revealed the presence of highly fragmented DNA below 200 bp in length, which are not expected to interfere with cultured cells^35^. Analysis of the cytocompatibility of the PanMa hydrogel confirmed that the residual DNA did not impaired cellular viability.

Similar to a previous study, we observed a destruction of the laminin network in the solid PanMa scaffold^33^. According to our data, laminin degradation does not result from the decellularization agent, but is due to self-digestion of the pancreas, most likely because of liberated enzymes from lysed acinar cells. This assumption is corroborated by the fact that an intact laminin ECM was found in SISser and LungMa, although both scaffolds were produced with the same decellularization reagent. Acinar cells produce several digestive enzymes that upon activation can cleave ECM peptides. Elebring et al. achieved preservation of laminin during decellularization of porcine pancreas using phenylmethylsulfonyl fluoride (PMSF)^43^. A comparative study of Gaetani et al. further showed that initial treatment of the porcine pancreas with 0.1 mM protease inhibitor Gabexate for 30 min had a beneficial effect on the retention of the basal laminar components laminin and collagen IV^44^. In contrast to porcine tissue, protease inhibition does not seem to be necessary for ECM preservation in rodent and human tissue as studies also demonstrate proper ECM retention without protease inhibitor treatment^31,32,45–47^. Although the laminin network was not intact, we verified the presence of different laminin subunits in the PanMa using mass-spectrometry in a previous study^33^. This suggests that laminin might still be an active signalling peptide in the PanMa hydrogel.

HEC-containing spheroids cultured in the PanMa hydrogel or Matrigel underwent morphological changes in terms of spheroid shape and distribution of hormone^+^ cells within the spheroids. In detail, we observed a reduced demarcation of the spheroids 14 days after encapsulation. Live/Dead staining proved that this is not due to a shedding of dead cells, suggesting that cells start emigrating from the spheroid. This was observed in both Matrigel and the PanMa hydrogel, demonstrating that this could be a general behavior of pancreatic endocrine cells after long-term culture in a hydrogel. Jiang et al. reported a sprouting of rat and human islet cells shortly after islet encapsulation in a bladder- or pancreas-derived ECM hydrogel, which were characterized as CD31^+^/Insulin^-^/CD44^-^/CD105^-^/CD90^-^ islet cells^48^. Whether such cell types are present in the hiPSC-derived endocrine spheroids and match the emigrating cells remains unclear. Apart from this, we observed an increased number of CPEP^+^ cells in the center of the spheroids after 7 days of culture in the hydrogels as well as in suspension, suggesting a changing cytoarchitecture with ongoing culture independent of hydrogel culture. Other groups observed a change in the cytoarchitecture of human islets upon islet isolation, resulting in an increased ratio of β-cells in the peripheral zone of the islet^32,49^. Interestingly, 7 days of culture in a human pancreas ECM-derived hydrogel prevented this effect and showed higher numbers of β-cells in the islet center, similar to that of native islets^32^. Together, the data from our study and other groups^32,49^ suggest an equal β-cell distribution within the islet as a sign for islet differentiation, in case of humans. In future studies, it would be interesting to investigate a possible correlation between islet cytoarchitecture and islet state in terms of viability, dedifferentiation and function.

Other groups have reported a beneficial effect of the ECM on survival and insulin secretion of primary islet cells, such as MIN6 cells^50^, as well as primary mouse^51^, rat^48^ and human islets^48,52–54^. Based on this, we investigated whether the ECM can also improve the β-cell identity of immature hiPSC-derived endocrine cells. Differentiated β-cells are expected to exhibit a certain expression pattern, including PDX1, NKX6.1, MAFA and INS/CPEP, and being negative for other hormones like SST and GCG^55^. HECs were generated according to the 4-stage protocol by Rezania et al.^2^ followed by aggregate formation and suspension culture for 7 days^3^. The obtained cells represent HECs with an immature endocrine phenotype^2,3^, indicated by a missing co-localization of PDX1/NK6.1/MAFA and CPEP as well as a polyhormonal character. In contrast to the original publication reporting 30% NKX6.1^+^/CPEP^+^ cells by the end of differentiation (day 21 in the original protocol), we could not detect NKX6.1^+^/CPEP^+^ cells at this time point of differentiation (day 21 of differentiation in the original protocol equals CTRL day 0 in this study). Further culture resulted in only few NKX6.1^+^/CPEP^+^ cells. The low yield of NKX6.1^+^/CPEP^+^ might be explained by the use of a hiPSC line instead of human embryonic SCs (hESCs), as used in the original publication. Studies have shown that not only the presence, but the sequential expression of single factors is of utmost importance for the establishment of β-cell identity. Onset of *NKX6.1* expression after endocrinogenesis, marked by the transient expression of *NGN3*, results in polyhormonal cells, which later mostly convert to GCG^+^ α-like cells^8,56,57^. This corresponds to the increasing GCG^+^ and decreasing CPEP^+^ area we observed with ongoing culture. Interestingly, encapsulation in Matrigel prevented this trend and resulted in less GCG^+^ area and a higher frequency of MAFA^+^ cells. This suggests that Matrigel induces a GCG-repressive effect by promoting expression of the β-cell specific maturation factor MAFA. However, HECs in Matrigel did not exhibit changes in CPEP^+^ area, demonstrating that Matrigel does not improve β-cell identity. Culture in the PanMa hydrogel led to a stable expression of β-cell markers for 7 days but did not promote the differentiation of hiPSC-derived HECs. Thus, we cannot confirm that the positive effect of the ECM on mature primary islets^48,51–54^ can be translated to immature hiPSC-derived islets.

Considering the biological and physical characteristics of the ECM, there are several parameters that can potentially influence pancreatic cell differentiation. ECM protein composition defines the physical and structural properties of the ECM, but also has a direct influence on cellular behavior. Singh et al. recently demonstrated an increased stimulation index of hESC-derived endocrine cells when cultured on individual ECM components such as laminin 511, Collagen IV or Fibronectin^58^. Although this study is hardly comparable to our study due to the use of hESCs, 2D monolayer culture and different differentiation protocols, it suggests that the PanMa might lack ECM proteins that are important for the promotion of β-cell function. Moreover, some ECM components contained in the PanMa hydrogel and the Matrigel might have adverse effects on endocrine maturation. Indeed, Kaido et al. have shown a decrease in insulin gene expression in human fetal and adult β-cells cultured on Collagen IV and Vitronectin^17^. Thus, future studies should focus on the composition of derived hydrogels to exclude an excessive proportion of ECM components with an undesired effect. Next to ECM composition, the rheological properties of the ECM can influence cellular behaviour. With regard to pancreatic development, studies have shown that cell shape, the state of the actin-cytoskeleton and transduced mechanical forces are decisive for the effectiveness of endocrinogenesis during in vitro differentiation^56,59^. Regulating these factors via substrate stiffness revealed an improved endocrinogenesis on soft substrates by promoting a compact cell shape. The used PanMa hydrogel exhibited a storage modulus of 200–400 Pa, which can be considered as soft in comparison to other tissues, such as liver (2 kPa) or kidney (4–8 kPa)^60^, or cell culture substrates (PET membrane: 2–2.7 GPa)^61^. Storage moduli of the PanMa hydrogel are slightly lower than that of native porcine (900 Pa) and human pancreas (636–1166 Pa)^62^ and slightly higher compared to decellularized porcine pancreas (89 Pa)^33^, demonstrating that the PanMa hydrogel approximately recapitulates pancreas tissue stiffness. An influencing factor of ECM composition and physical properties is ageing. In this study, we used pancreatic tissue from 6 to 10 week old piglets. Studies with decellularized rat and human liver show that age-signatures are preserved in decellularized scaffolds and affect the function of primary liver cells seeded on the scaffold^63^. In mammals, β-cell maturation is known to occur postnatally and continues post-weaning^55^. Therefore, it is conceivable that the young age of the pancreatic tissue in this study might exhibit a signalling profile promoting a rather immature phenotype instead of promoting pancreatic maturation. In this case, it would be interesting to investigate potential age-related differences of pancreatic ECM and to test the effects of a more mature pancreatic ECM scaffold in the experimental set-up of this study.

## Conclusion

In the present study, we demonstrate the production of a pancreas specific ECM hydrogel from whole organ decellularized porcine pancreas. The PanMa hydrogel supports the encapsulation and culture of hiPSC-derived HECs. We further show that the PanMa hydrogel enables the retention of β-cell specific gene expression during 7 days of culture. Long-term culture of HECs in the PanMa hydrogel or Matrigel does not improve β-cell identity, suggesting that encapsulation in an ECM hydrogel is not sufficient to trigger endocrine development of hiPSC-derived HECs. In conclusion, our study provides a basis for ECM hydrogels that are more adapted to the demands of SC-BCs.

## Methods

### Animal care

Animal research was approved by the Ethics Committee of the District of Lower Franconia, Würzburg, Germany (approval number: 55.2-2532-2-256 and 55.2.2-2532-2-1477-27). Care of the animals was in accordance with the Guide for Care and Use of Laboratory Animals published by the National Institute of Health (NIH publication no. 85e23, revised 1996) and approved by the institutional board of animal protection (Department for animal welfare, University of Würzburg). The process of organ explantation was performed in compliance with the German Animal Protection Law (§4 Abs.3) with regular notification of the responsible authorities by the animal protection officer. Animals were included in the study if they were in healthy conditions at the time point of anesthesia and if organs showed a normal anatomy. The study is reported in accordance with the ARRIVE guidelines.

### Organ explantation and decellularization

6–10 week old piglets (male and female, n = 10) were narcotized by the injection of Stresnil® (3 mg/kg body weight; Elanco) and Ursotamin^®^ (14 mg/kg body weight; Serumwerk). Subsequently, heparin (500–600 Units/kg body weight, Ratiopharm^®^) was infused intravenously to prevent blood coagulation. 15 min after heparinization, piglets were euthanized by infusion of T61 (0.5 ml/kg body weight, MSD Animal Health).

Pancreas explantation and preparation was conducted as described before^33^. Whole organ perfusion was accomplished via accesses to the *splenic artery* and the *pancreatic duct*.

#### PanMa standard protocol

For the production of ECM hydrogels, PanMas were produced according to our previously published decellularization protocol^33^.

#### PanMa shortened protocol

Pancreata were perfused via the *splenic artery* and the *pancreatic duct* with Milli-Q^®^ H_2_O (2 h), Sodium deoxycholate (30970, Sigma-Aldrich) (4 h), Phosphate Buffered Saline Solution without CaCl_2_ and MgCl_2_ (PBS^-^, D8537, Sigma-Aldrich) (16 h), Sodium deoxycholate (4 h), PBS^-^ (24 h) and PBS^-^ with 1× Penicillin/Streptomycin (P/S, P4333, Sigma-Aldrich) (48 h). All perfusion steps were performed at a constant flow rate of 3.9 ml/min. Next, pancreata were disconnected from the peristaltic pumps, transferred to a beaker glass and treated with 1 mg/ml DNAse (10104159001, Roche) dissolved in PBS with CaCl_2_ and MgCl_2_ (PBS^+^, D8662, Sigma-Aldrich) at 37 °C for 16 h.

#### SISser and LungMa

SISser and LungMa were generated according to previously published protocols by decellularization of jejunal segments or lung tissue, respectively^33,64^.

Decellularized scaffolds were stored in PBS^-^ with 1× P/S at 4 °C with daily liquid exchange. For the production of ECM hydrogels, scaffolds were frozen at − 80 °C until further use. Importantly, we observed that ECM hydrogels could not be produced from γ-irradiated scaffolds due to impaired gelling. Therefore, γ-irradiation was only used for long-term storage of ECM-scaffolds that were not intended for hydrogel production. γ-irradiation was performed with a dosage of > 25 kG by the sterilization service from BBF steriXpert (Kernen-Rommelshausen, Germany). Sterilized scaffolds were stored in PBS^-^ at 4 °C.

### DNA content

Samples for DNA extraction were taken either from native tissue or the generated ECM scaffolds from different parts of the organ. Total DNA was extracted from native tissue and decellularized samples using the DNEasy Blood and Tissue Kit (69506, Qiagen). For this, 3 mg of lyophilized tissue were digested with Proteinase K at 56 °C overnight and DNA was purified as instructed by the manufacturer. Quantification of extracted DNA was conducted with the Quant-iT™ PicoGreen™ dsDNA Assay Kit (P11496, Invitrogen) following the manufacturer’s guidelines. The fluorescence intensity at 480 nm excitation and 525 nm emission was determined using an Infinite M200 Plate Reader (TECAN) and the DNA content was calculated from a standard curve.

### Hydrogel production

For the production of pancreas specific ECM hydrogels, only PanMas generated by the standard protocol without γ-irradiation were used. First, PanMas were frozen at − 80 °C and subsequently lyophilized using an Alpha 1–2 LO Plus lyophilizer (Christ). Lyophilized PanMas were crushed into powder using a Tissue rupture (9002755, Qiagen) equipped with a steel probe (9017341, Qiagen), and the obtained powder was strained through a polyester mesh with a mesh size of 500 µm. The strained PanMa powder was digested according to previously published protocols to obtain a pregel^26^. In detail, 10 mg of sterilized PanMa hydrogel was dissolved in 1 ml digest buffer (0.1% Pepsin (77160, Sigma-Aldrich) in 0.1 N HCl (K025.1, Carl Roth)) and stirred for 72 h at RT. PanMa pregels were centrifuged at 14,000*g* at 4 °C for 15 min to remove insoluble ECM components. Subsequently, the PanMa pregels were neutralized by the addition of 1/9 (v/v) 10× PBS^-^ (D1283, Sigma-Aldrich) as well as 1/10 (v/v) 0.1 M NaOH (1.09137, Supelco) and placed at 37 °C, 95% humidity, 5% CO_2_ for 15–30 min. Supernatants of the obtained pregels could be stored at − 20 °C for 2 years without apparent effects on gelling behaviour.

### Silver staining

Pregels were diluted in digest solution to a final protein concentration of 100 µg/ml and neutralized by addition of 1/10 (v/v) 0.1 M NaOH. After mixing with Laemmli Buffer, samples were incubated at 95 °C for 5 min and loaded on a sodium dodecyl sulfate polyacrylamide gel. Electrophoresis was performed at 25 mA and 400 V. For silver staining, the gel was incubated on a rocking shaker in the following solutions: 5 min Milli-Q^®^ H_2_O containing 8.6 M acetone (T906.1, Carl Roth), 76.5 mM trichloroacetic acid (7437.1, Carl Roth) and 5.1 mM formaldehyde (1.04003.1000, Sigma), three times wash in Milli-Q^®^ H_2_O, 5 min Milli-Q^®^ H_2_O, three times wash in Milli-Q^®^ H_2_O, 5 min Milli-Q^®^ H_2_O containing 8.6 M acetone, 1 min Milli-Q^®^ H_2_O containing 1.1 mM sodium thiosulfate (106516, Supelco), three times wash in Milli-Q^®^ H_2_O, 8 min Milli-Q^®^ H_2_O containing 15.7 mM silver nitrate (209139, Supelco) and 123.2 mM formaldehyde, five times wash in Milli-Q^®^ H_2_O for 5 min each. Subsequently, the staining was developed by incubation in Milli-Q^®^ H_2_O containing 0.3 mM sodium thiosulfate, 5.1 mM formaldehyde and 188.7 mM sodium carbonate (A135.1, Carl Roth). The reaction was stopped by incubation in Milli-Q^®^ H_2_O with 166.5 mM acetic acid (6755.2, Carl Roth) and the gel was imaged.

### Rheology

Rheological measurements were performed using an Anton Parr MCR 301 rheometer equipped with a 25 mm diameter parallel plate. The supernatant of ECM digests was neutralized and the pregel loaded onto the rheometer. Measurements were performed with a plate-to-plate gap of 0.3 mm, an angular frequency of 10 rad/s and a sinusoidal strain with an amplitude of 0.1%. During measurements, the gels were subjected to a temperature sweep (5 to 37 °C) with an increment rate of 0.05 °C/s. After reaching 37 °C, the temperature was kept at 37 °C for 100 min.

### hiPSC maintenance culture

IMR90-4 hiPSCs (WiCell) were cultured on plates coated with Matrigel (356231, Corning) or Geltrex (A1413302, Gibco) in mTeSR1 medium (85850, Stemcell Technologies). No difference was observed between cultures maintained on Matrigel or Geltrex regarding pluripotency or differentiation capacity. hiPSCs were passaged at 60% confluence at a 1:6–1:20 split ratio using Gentle Cell dissociation reagent (100-0485, Stemcell Technologies) according to the manufacturer’s guidelines. Passaged cells were plated in mTeSR1 medium containing 10 µM Y-27632 (1254, Tocris) for the first 24 h. hiPSC maintenance cultures were routinely tested for mycoplasma via PCR and stem cell marker expression using flow cytometry (Fig. S5).

### Generation of hiPSC-derived hormone expressing pancreatic endocrine cells

HECs were generated according to the 4-stage protocol by Rezania et al. with small adaptions followed by 7 days in suspension culture^2,3^. Briefly, IMR90-4 cells were detached as single cells by incubation with Accutase (A6964, Sigma-Aldrich) for 5 min at 37 °C. Cells were seeded at a density of 1.04 × 10^5 ^cells/cm^2^ in mTeSR1 medium supplemented with 10 µM Y-27632 on plates coated with Matrigel or Geltrex. No influence of the coating material on the differentiation was observed. 24 h after seeding, differentiation was induced using the following media compositions: Stage 1, day 1: RPMI medium with Glutamax (61870010, Gibco) supplemented with 0.2% (v/v) FCS (FCS.ADD.0500, Bio & Sell), 100 ng/ml Activin A (120-14E, Peprotech) and 2 µM CHIR99021 (Cay-13122, Biomol). Stage 1, day 2–3: RPMI medium with Glutamax supplemented with 0.5% (v/v) FCS and 100 ng/ml Activin A. Stage 2, day 4–6: DMEM/F12 with Glutamax (31331028, Gibco) supplemented with 2% (v/v) FCS and 50 ng/ml FGF7 (100–19, Peprotech). Stage 3, day 7–10: DMEM high glucose with Glutamax (61965026, Gibco) supplemented with 1% (v/v) B27 without Vitamin A (12587010, Gibco), 100 ng/ml Noggin (AF-250-38, Peprotech), 250 nM Sant-1 (S4572, Sigma-Aldrich) and 2 µM Retinoic Acid (R2625, Sigma-Aldrich). Stage 4, day 11–14: DMEM high glucose with Glutamax supplemented with 1% (v/v) B27 without Vitamin A, 100 ng/ml Noggin, 1 µM ALK5 inhibitor II (ALX-270-445, Enzo Life Sciences) and 50 nM TPPB (5343, Tocris). At day 14, cells were transferred to suspension culture. To this purpose, cells were incubated with 5 mg/ml Dispase (17105041, Gibco) dissolved in DMEM high glucose with Glutamax at 37 °C for 5–7 min. As soon as the edges of the cell layer lifted, cells were rinsed with DMEM high glucose with Glutamax twice and collected in Stage 5 medium: DMEM high glucose with Glutamax supplemented with 1% (v/v) B27 without Vitamin A, 1 µM T3 (T6397, Sigma-Aldrich), 10 µg/ml Heparin (H3149, Sigma-Aldrich), 10 µM ALK5 inhibitor II, 10 nM γ-secretase inhibitor XX (565789, Sigma-Aldrich) and LDN193189 (72146, Stemcell Technologies). Cells were fragmented manually by careful pipetting and transferred to 6-well plates treated with Anti-Adherence Rinsing Solution (07010, StemCell Technologies). Spheroids were cultured for 7 days in Stage 5 medium with daily medium change. P/S was added to the medium not before stage 3, as we observed adverse cellular effects at earlier time points.

### Encapsulation of hiPSC-derived hormone expressing pancreatic endocrine cells

HECs were collected at day 21 of differentiation (end of Stage 5) in Stage 5 medium, counted and encapsulated in drops of 75% PanMa hydrogel or 50% Matrigel. For encapsulation in 75% PanMa hydrogel, 600–1000 HECs in 100 µl Stage 5 medium were transferred to a centrifuge tube. 300 µl of neutralized, pre-cooled (4 °C) PanMa pregel were added to achieve a 75% PanMa hydrogel with a final concentration of 150–250 spheroids/100 µl. The spheroid suspension was mixed carefully and drops of 10 µl containing 15–25 spheroids were plated in pre-warmed (37 °C) 24-well cell-culture plates. After 15–30 min incubation at 37 °C, 95% humidity, 5% CO_2_, PanMa hydrogel drops were immersed in Stage 5 medium. For encapsulation in 50% Matrigel, 300–500 HECs were placed in 100 µl Stage 5 medium in a centrifuge tube and mixed with 100 µl pre-cooled (4 °C) Matrigel. This resulted in a 50% Matrigel mixture with a final concentration of 150–250 HECs/100 µl. Drops of 10 µl each containing 15–25 spheroids were plated in pre-warmed (37 °C) 24-well cell-culture plates. After 5–10 min incubation at 37 °C, 95% humidity, 5% CO_2_, Matrigel drops were immersed in Stage 5 medium.

Encapsulated HECs were cultured for 21 days with daily medium change (Stage 5 medium). HECs cultured in suspension were used as a control. Brightfield images of HECs cultured in suspension, in the PanMa hydrogel or in Matrigel were taken with an EVOS XL digital microscope (Thermofisher).

### Fluorescein-diacetate (FDA)/propidium iodide (PI) staining

HECs cultured in suspension or encapsulated in the PanMa hydrogel or Matrigel were rinsed with PBS^-^ and incubated for 10–20 s with PBS^−^ supplemented with 0.5 µg/ml FDA (F7378, Sigma-Aldrich) and 0.5 µg/ml PI (P4170, Sigma-Aldrich). The FDA/PI solution was removed and HECs were washed with PBS^-^ and imaged immediately. FDA/PI treated HECs were imaged with a BZ-9000 fluorescent microscope (Keyence) with a GFP filter (excitation: 470/40 nm) for FDA and a TRITC filter (excitation: 545/25 nm) for PI. For quantification, the fluorescent signal of FDA and PI was individually quantified from a total of 5–12 images of three biological replicates for each condition using an image J macro (Supplementary Table 2, Macro #11 and #12). Cell death was given as the PI area to FDA area ratio.

### RT-qPCR

Encapsulated HECs were removed from the PanMa hydrogel or Matrigel by pipetting and collected. Total RNA was isolated using the RNeasy Micro Kit (74004, Qiagen) according to the manufacturer’s guidelines including DNA digestion (79254, Qiagen). Isolated RNA was quantified using a NanoQuant Plate (Tecan) in combination with an Infinite M200 Plate Reader (Tecan) and 500 ng RNA were used for cDNA synthesis carried out using the iScript™ cDNA Synthesis Kit (1708891, Bio-Rad) according to the manufacturer’s instructions. RT-qPCR was performed using the SsoFast EvaGreen Supermix (1725201, Bio-Rad) and a CFX 96 Touch™ Real-Time PCR Detection System (Bio-Rad). All reactions were carried out in duplicates with an annealing temperature of 60 °C. Plates were designed using the sample maximation method. The obtained data were analyzed according to the ΔΔCT-method with RPL4 and RPL6 as housekeeping genes. The following primer sequences were used:

**Table:** 

Gene/RefSeq	Forward primer	Reverse primer	Reference
*GCG* NM_002054.5	AAGCATTTA CTTTGTGGCTGGATT	TGATCTGGATTTCTCCTCTGTGTCT	D’amour et al.^1^
*INS* NM_000207.3	GCAGCCTTTGTGAACCAACAC	CCCCGCACACTAGGTAGAGA	Zhang et al.^65^
*MAFA* NM_201589.4	CTTCAGCAAGGAGGAGGTCATC	CTCGTATTTCTCCTTGTACAGGTCC	Zhang et al.^65^
*NKX6.1* NM_006168.3	AGACCCACTTTTTCCGGACA	CCAACGAATAGGCCAAACGA	Zhang et al.^65^
*PDX1* NM_000209.4	AAGTCTACCAAAGCTCACGCG	GTAGGCGCCGCCTGC	D’amour et al.^1^
*RPL4* NM_000968.4	GCCTGCTGTATTCAAGGCTC	GGTTGGTGCAAACATTCGGC	–
*RPL6* NM_001024662	ATTCCCGATCTGCCATGTATTC	TACCGCCGTTCTTGTCACC	–
*SST* NM_001048.4	CCCAGACTCCGTCAGTTTCT	ATCATTCTCCGTCTGGTTGG	Zhang et al.^65^

### Immunohistochemistry

Native and decellularized tissue samples were fixed in 4% Histofix (P087.3, Carl Roth) at 4 °C overnight and subsequently embedded in paraffin. Encapsulated HECs were mechanically released from the PanMa hydrogel or Matrigel by pipetting. The collected HECs were incubated with 4% Histofix for 30 min at 4 °C, washed two times with PBS^−^, and embedded in Histogel (HG-4000-012, Thermofisher), prior to paraffin embedding. 5 µm sections were prepared and stored at 37 °C overnight. Immunohistochemical stainings were performed as described previously^33^. In brief, dewaxed and rehydrated samples were incubated for 15 min in citrate buffer pH 6.0 at 100 °C for antigen retrieval. Subsequently, samples were transferred to PBS^-^ with 0.5% Tween (PBST) and treated with PBST containing 5% BSA (1126GR500, Biofroxx) for 30 min at RT prior to staining with primary antibodies diluted in blocking buffer at the following dilutions: 1:200 rabbit anti-laminin (ab11575, Abcam), 1:300 rat anti-C-Peptide (GN-ID4-S, Developmental Studies Hybridoma Bank (DSHB)), 1:1000 mouse anti-glucagon (ab10988, Abcam), 1:250 rabbit anti-somatostatin (HPA019472, Sigma-Aldrich), 1:250 goat anti-PDX1 (AF2419, R&D), 1:250 goat-anti NKX6.1 (AF5857, R&D), 1:250 rabbit anti-MAFA (ab26405, Abcam). Immunolabeled samples were washed three times for 5 min with PBST and incubated with secondary antibodies diluted 1:400 in antibody dilution solution for 2 h at RT. After three times washing with PBST for 5 min each, samples were mounted with Fluoromount G containing DAPI (00-4959-52, Invitrogen). Images of immunofluorescent stainings were acquired with a Keyence BZ-X810 using high resolution mode (PDX1, MAFA, CPEP, GCG, SST) or standard resolution (NKX6.1) in combination with the full focus mode. For quantification, ≥ 15 spheroids of ≥ 3 biological replicates were analyzed using image J macros (Supplementary Table 2, Macro #1–#10). At this, single channels were quantified separately using the respective macro. The proportion of marker-positive nuclei was determined by quantification of positive nuclei in relation to the total number of DAPI^+^ objects. Quantification of pancreatic hormones was achieved by measuring the area of the fluorescent signal for each hormone followed by normalization to DAPI^+^ signal area. If images contained several spheroids or non-relevant objects (dirt, air bubbles, necrotic cores), individual spheroids were excised manually prior to quantification. For cropping of individual spheroids the same mask was used for all channels of the image.

### Image processing

For presentation in this paper, acquired images were processed with Fiji using the following operations: subtract background (rolling ball diameter), adjust brightness and contrast, cropping, split channels, merge channels, stack to RGB, insert scale bar.

### Statistics

Statistical analyses were carried out with GraphPad Prism (version 9.5.1). Normality of the data from biological samples was assumed, but not tested due to low sample size. Accordingly, data sets were analyzed with parametric tests (One-way ANOVA with Tukey’s or Sidak’s multiple comparisons). All statistical tests were carried out with a 95% confidence interval. P-values are depicted as the following: P > 0.05, *P < 0.05, **P < 0.01, ***P < 0.001, ****P < 0.0001. Detailed information of the statistical tests, the replicate size, the compared groups and exact p-values are given in supplementary Table 1.

### Institutional review board statement

The animal study protocol was approved by the Ethics Committee of the District of Lower Franconia, Würzburg, Germany (approval number: 55.2-2532-2-256, date: 22.07.2017) and recently extended (approval number: 55.2.2-2532-2-1477-27, date: 03.05.2022).

## Supplementary Information

Supplementary Information.

## Data Availability

All data can be provided by the authors upon request. Please contact the corresponding author (constantin.berger@uni-wuerzburg.de) for data requests.

## References

[1] D’Amour, K. A. *et al.* Production of pancreatic hormone-expressing endocrine cells from human embryonic stem cells. *Nat. Biotechnol.* **24**, 1392–1401. 10.1038/nbt1259 (2006). 17053790 10.1038/nbt1259

[2] Rezania, A. *et al.* Maturation of human embryonic stem cell-derived pancreatic progenitors into functional islets capable of treating pre-existing diabetes in mice. *Diabetes* **61**, 2016–2029. 10.2337/db11-1711 (2012). 22740171 10.2337/db11-1711 PMC3402300

[3] Rezania, A. *et al.* Reversal of diabetes with insulin-producing cells derived in vitro from human pluripotent stem cells. *Nat. Biotechnol.* **32**, 1121–1133. 10.1038/nbt.3033 (2014). 25211370 10.1038/nbt.3033

[4] Pagliuca, F. W. *et al.* Generation of functional human pancreatic β cells in vitro. *Cell* **159**, 428–439. 10.1016/j.cell.2014.09.040 (2014). 25303535 10.1016/j.cell.2014.09.040 PMC4617632

[5] Nair, G. G. *et al.* Recapitulating endocrine cell clustering in culture promotes maturation of human stem-cell-derived β cells. *Nat. Cell Biol.* **21**, 263–274. 10.1038/s41556-018-0271-4 (2019). 30710150 10.1038/s41556-018-0271-4 PMC6746427

[6] Hogrebe, N. J., Maxwell, K. G., Augsornworawat, P. & Millman, J. R. Generation of insulin-producing pancreatic β cells from multiple human stem cell lines. *Nat. Protoc.*10.1038/s41596-021-00560-y (2021). 34349281 10.1038/s41596-021-00560-y PMC8529911

[7] Du, Y. *et al.* Human pluripotent stem-cell-derived islets ameliorate diabetes in non-human primates. *Nat. Med.* **28**, 272–282. 10.1038/s41591-021-01645-7 (2022). 35115708 10.1038/s41591-021-01645-7

[8] Veres, A. *et al.* Charting cellular identity during human in vitro β-cell differentiation. *Nature* **569**, 368–373. 10.1038/s41586-019-1168-5 (2019). 31068696 10.1038/s41586-019-1168-5 PMC6903417

[9] Augsornworawat, P., Maxwell, K. G., Velazco-Cruz, L. & Millman, J. R. Single-cell transcriptome profiling reveals β cell maturation in stem cell-derived islets after transplantation. *Cell Rep.* **32**, 108067. 10.1016/j.celrep.2020.108067 (2020). 32846125 10.1016/j.celrep.2020.108067 PMC7491368

[10] Agulnick, A. D. *et al.* Insulin-producing endocrine cells differentiated in vitro from human embryonic stem cells function in macroencapsulation devices in vivo. *Stem Cells Transl. Med.* **4**, 1214–1222. 10.5966/sctm.2015-0079 (2015). 26304037 10.5966/sctm.2015-0079 PMC4572906

[11] Kroon, E. *et al.* Pancreatic endoderm derived from human embryonic stem cells generates glucose-responsive insulin-secreting cells in vivo. *Nat. Biotechnol.* **26**, 443–452. 10.1038/nbt1393 (2008). 18288110 10.1038/nbt1393

[12] In vitro and in vivo studies. de Carlo. Pancreatic acellular matrix supports islet survival and function in a synthetic tubular device. *Int J Mol Med* **25**, 195–202. 10.3892/ijmm_00000330 (2009). 10.3892/ijmm_00000330 20043127

[13] Musah, S. *et al.* Glycosaminoglycan-binding hydrogels enable mechanical control of human pluripotent stem cell self-renewal. *ACS Nano* **6**, 10168–10177. 10.1021/nn3039148 (2012). 23005914 10.1021/nn3039148 PMC3509190

[14] Engler, A. J., Sen, S., Sweeney, H. L. & Discher, D. E. Matrix elasticity directs stem cell lineage specification. *Cell* **126**, 677–689. 10.1016/j.cell.2006.06.044 (2006). 16923388 10.1016/j.cell.2006.06.044

[15] Thomas, F. T. *et al.* Anoikis, extracellular matrix, and apoptosis factors in isolated cell transplantation. *Surgery* **126**, 299–304. 10.1016/S0039-6060(99)70169-8 (1999). 10455898 10.1016/S0039-6060(99)70169-8

[16] Ris, F. *et al.* Impact of integrin-matrix matching and inhibition of apoptosis on the survival of purified human beta-cells in vitro. *Diabetologia* **45**, 841–850. 10.1007/s00125-002-0840-7 (2002). 12107728 10.1007/s00125-002-0840-7

[17] Kaido, T. *et al.* Impact of defined matrix interactions on insulin production by cultured human beta-cells: Effect on insulin content, secretion, and gene transcription. *Diabetes* **55**, 2723–2729. 10.2337/db06-0120 (2006). 17003336 10.2337/db06-0120

[18] Nyitray, C. E., Chavez, M. G. & Desai, T. A. Compliant 3D microenvironment improves β-cell cluster insulin expression through mechanosensing and β-catenin signaling. *Tissue Eng. Part A* **20**, 1888–1895. 10.1089/ten.TEA.2013.0692 (2014). 24433489 10.1089/ten.TEA.2013.0692 PMC4085995

[19] Gan, W. J. *et al.* Local integrin activation in pancreatic β cells targets insulin secretion to the vasculature. *Cell Rep.* **24**, 2819-2826.e3. 10.1016/j.celrep.2018.08.035 (2018). 30208309 10.1016/j.celrep.2018.08.035

[20] Kuehn, C., Dubiel, E. A., Sabra, G. & Vermette, P. Culturing INS-1 cells on CDPGYIGSR-, RGD- and fibronectin surfaces improves insulin secretion and cell proliferation. *Acta Biomaterialia* **8**, 619–626. 10.1016/j.actbio.2011.10.036 (2012). 22085924 10.1016/j.actbio.2011.10.036

[21] Saldin, L. T., Cramer, M. C., Velankar, S. S., White, L. J. & Badylak, S. F. Extracellular matrix hydrogels from decellularized tissues: Structure and function. *Acta Biomaterialia* **49**, 1–15. 10.1016/j.actbio.2016.11.068 (2017). 27915024 10.1016/j.actbio.2016.11.068 PMC5253110

[22] Voytik-Harbin, S. L., Brightman, A. O., Waisner, B. Z., Robinson, J. P. & Lamar, C. H. Small intestinal submucosa: A tissue-derived extracellular matrix that promotes tissue-specific growth and differentiation of cells in vitro. *Tissue Eng.* **4**, 157–174. 10.1089/ten.1998.4.157 (1998). 10.1089/ten.1998.4.157

[23] Giobbe, G. G. *et al.* Extracellular matrix hydrogel derived from decellularized tissues enables endodermal organoid culture. *Nat. Commun.* **10**, 5658. 10.1038/s41467-019-13605-4 (2019). 31827102 10.1038/s41467-019-13605-4 PMC6906306

[24] Freytes, D. O., Martin, J., Velankar, S. S., Lee, A. S. & Badylak, S. F. Preparation and rheological characterization of a gel form of the porcine urinary bladder matrix. *Biomaterials* **29**, 1630–1637. 10.1016/j.biomaterials.2007.12.014 (2008). 18201760 10.1016/j.biomaterials.2007.12.014

[25] Singelyn, J. M. *et al.* Naturally derived myocardial matrix as an injectable scaffold for cardiac tissue engineering. *Biomaterials* **30**, 5409–5416. 10.1016/j.biomaterials.2009.06.045 (2009). 19608268 10.1016/j.biomaterials.2009.06.045 PMC2728782

[26] Wolf, M. T. *et al.* A hydrogel derived from decellularized dermal extracellular matrix. *Biomaterials* **33**, 7028–7038. 10.1016/j.biomaterials.2012.06.051 (2012). 22789723 10.1016/j.biomaterials.2012.06.051 PMC3408574

[27] DeQuach, J. A., Yuan, S. H., Goldstein, L. S. B. & Christman, K. L. Decellularized porcine brain matrix for cell culture and tissue engineering scaffolds. *Tissue Eng. Part A* **17**, 2583–2592. 10.1089/ten.TEA.2010.0724 (2011). 21883047 10.1089/ten.TEA.2010.0724 PMC3204197

[28] Nagao, R. J. *et al.* Decellularized human kidney cortex hydrogels enhance kidney microvascular endothelial cell maturation and quiescence. *Tissue Eng. Part A* **22**, 1140–1150. 10.1089/ten.TEA.2016.0213 (2016). 27481445 10.1089/ten.TEA.2016.0213 PMC5073226

[29] Saheli, M. *et al.* Three-dimensional liver-derived extracellular matrix hydrogel promotes liver organoids function. *J. Cell. Biochem.* **119**, 4320–4333. 10.1002/jcb.26622 (2018). 29247536 10.1002/jcb.26622

[30] Pouliot, R. A. *et al.* Porcine lung-derived extracellular matrix hydrogel properties are dependent on pepsin digestion time. *Tissue Eng. Part C Methods* **26**, 332–346. 10.1089/ten.TEC.2020.0042 (2020). 32390520 10.1089/ten.TEC.2020.0042 PMC7310225

[31] Sackett, S. D. *et al.* Extracellular matrix scaffold and hydrogel derived from decellularized and delipidized human pancreas. *Sci. Rep.* **8**, 10452. 10.1038/s41598-018-28857-1 (2018). 29993013 10.1038/s41598-018-28857-1 PMC6041318

[32] Tremmel, D. M. *et al.* A human pancreatic ECM hydrogel optimized for 3-D modeling of the islet microenvironment. *Sci. Rep.* **12**, 7188. 10.1038/s41598-022-11085-z (2022). 35504932 10.1038/s41598-022-11085-z PMC9065104

[33] Berger, C. *et al.* Matrix decoded - A pancreatic extracellular matrix with organ specific cues guiding human iPSC differentiation. *Biomaterials* **244**, 119766. 10.1016/j.biomaterials.2020.119766 (2020). 32199284 10.1016/j.biomaterials.2020.119766

[34] Kim, S. *et al.* Tissue extracellular matrix hydrogels as alternatives to Matrigel for culturing gastrointestinal organoids. *Nat. Commun.* **13**, 1692. 10.1038/s41467-022-29279-4 (2022). 35354790 10.1038/s41467-022-29279-4 PMC8967832

[35] Crapo, P. M., Gilbert, T. W. & Badylak, S. F. An overview of tissue and whole organ decellularization processes. *Biomaterials* **32**, 3233–3243. 10.1016/j.biomaterials.2011.01.057 (2011). 21296410 10.1016/j.biomaterials.2011.01.057 PMC3084613

[36] Hughes, C. S., Postovit, L. M. & Lajoie, G. A. Matrigel: A complex protein mixture required for optimal growth of cell culture. *Proteomics* **10**, 1886–1890. 10.1002/pmic.200900758 (2010). 20162561 10.1002/pmic.200900758

[37] Jennings, R. E. *et al.* Development of the human pancreas from foregut to endocrine commitment. *Diabetes* **62**, 3514–3522. 10.2337/db12-1479 (2013). 23630303 10.2337/db12-1479 PMC3781486

[38] Piper, K. *et al.* Beta cell differentiation during early human pancreas development. *J. Endocrinol.* **181**, 11–23. 10.1677/joe.0.1810011 (2004). 15072563 10.1677/joe.0.1810011

[39] Schaffer, A. E. *et al.* Nkx61 controls a gene regulatory network required for establishing and maintaining pancreatic Beta cell identity. *PLoS Genet.* **9**, e1003274. 10.1371/journal.pgen.1003274 (2013). 23382704 10.1371/journal.pgen.1003274 PMC3561089

[40] Bonal, C. & Herrera, P. L. Genes controlling pancreas ontogeny. *Int. J. Dev. Biol.* **52**, 823–835. 10.1387/ijdb.072444cb (2008). 18956314 10.1387/ijdb.072444cb

[41] Ma, Z. *et al.* Deciphering early human pancreas development at the single-cell level. *Nat. Commun.* **14**, 5354. 10.1038/s41467-023-40893-8 (2023). 37660175 10.1038/s41467-023-40893-8 PMC10475098

[42] Gouk, S.-S., Lim, T.-M., Teoh, S.-H. & Sun, W. Q. Alterations of human acellular tissue matrix by gamma irradiation: Histology, biomechanical property, stability, in vitro cell repopulation, and remodeling. *J. Biomed. Mater. Res. Part B Appl. Biomater.* **84**, 205–217. 10.1002/jbm.b.30862 (2008). 10.1002/jbm.b.30862 17497685

[43] Elebring, E., Kuna, V. K., Kvarnström, N. & Sumitran-Holgersson, S. Cold-perfusion decellularization of whole-organ porcine pancreas supports human fetal pancreatic cell attachment and expression of endocrine and exocrine markers. *J. Tissue Eng.* **8**, 2041731417738145. 10.1177/2041731417738145 (2017). 29118967 10.1177/2041731417738145 PMC5669317

[44] Gaetani, R. *et al.* Evaluation of different decellularization protocols on the generation of pancreas-derived hydrogels. *Tissue Eng. Part C Methods* **24**, 697–708. 10.1089/ten.TEC.2018.0180 (2018). 30398401 10.1089/ten.TEC.2018.0180 PMC6306687

[45] Goh, S.-K. *et al.* Perfusion-decellularized pancreas as a natural 3D scaffold for pancreatic tissue and whole organ engineering. *Biomaterials* **34**, 6760–6772. 10.1016/j.biomaterials.2013.05.066 (2013). 23787110 10.1016/j.biomaterials.2013.05.066 PMC3748589

[46] Napierala, H. *et al.* Engineering an endocrine neo-pancreas by repopulation of a decellularized rat pancreas with islets of Langerhans. *Sci. Rep.* **7**, 41777. 10.1038/srep41777 (2017). 28150744 10.1038/srep41777 PMC5288794

[47] Peloso, A. *et al.* The human pancreas as a source of protolerogenic extracellular matrix scaffold for a new-generation bioartificial endocrine pancreas. *Ann. Surg.* **264**, 169–179. 10.1097/SLA.0000000000001364 (2016). 26649588 10.1097/SLA.0000000000001364 PMC4882269

[48] Jiang, K. *et al.* 3-D physiomimetic extracellular matrix hydrogels provide a supportive microenvironment for rodent and human islet culture. *Biomaterials* **198**, 37–48. 10.1016/j.biomaterials.2018.08.057 (2019). 30224090 10.1016/j.biomaterials.2018.08.057 PMC6397100

[49] Lavallard, V. *et al.* Cell rearrangement in transplanted human islets. *FASEB J.* **30**, 748–760. 10.1096/fj.15-273805 (2016). 26534832 10.1096/fj.15-273805

[50] Beenken-Rothkopf, L. N. *et al.* The incorporation of extracellular matrix proteins in protein polymer hydrogels to improve encapsulated beta-cell function. *Ann. Clin. Lab. Sci.* **43**, 111–121 (2013). 23694784

[51] Stephens, C. H. *et al.* In situ type I oligomeric collagen macroencapsulation promotes islet longevity and function in vitro and in vivo. *Am. J. Physiol. Endocrinol. Metab.* **315**, E650–E661. 10.1152/ajpendo.00073.2018 (2018). 29894201 10.1152/ajpendo.00073.2018 PMC6230705

[52] Davis, N. E. *et al.* Enhanced function of pancreatic islets co-encapsulated with ECM proteins and mesenchymal stromal cells in a silk hydrogel. *Biomaterials* **33**, 6691–6697. 10.1016/j.biomaterials.2012.06.015 (2012). 22766242 10.1016/j.biomaterials.2012.06.015 PMC3701024

[53] Llacua, A., de Haan, B. J., Smink, S. A. & de Vos, P. Extracellular matrix components supporting human islet function in alginate-based immunoprotective microcapsules for treatment of diabetes. *J. Biomed. Mater. Res. Part A* **104**, 1788–1796. 10.1002/jbm.a.35706 (2016). 10.1002/jbm.a.35706 26990360

[54] Enck, K. *et al.* Effect of alginate matrix engineered to mimic the pancreatic microenvironment on encapsulated islet function. *Biotechnol. Bioeng.* **118**, 1177–1185. 10.1002/bit.27641 (2021). 33270214 10.1002/bit.27641 PMC8887826

[55] Barsby, T. & Otonkoski, T. Maturation of beta cells: Lessons from in vivo and in vitro models. *Diabetologia* **65**, 917–930. 10.1007/s00125-022-05672-y (2022). 35244743 10.1007/s00125-022-05672-y PMC9076740

[56] Hogrebe, N. J., Augsornworawat, P., Maxwell, K. G., Velazco-Cruz, L. & Millman, J. R. Targeting the cytoskeleton to direct pancreatic differentiation of human pluripotent stem cells. *Nat. Biotechnol.* **38**, 460–470. 10.1038/s41587-020-0430-6 (2020). 32094658 10.1038/s41587-020-0430-6 PMC7274216

[57] Sharon, N. *et al.* Wnt signaling separates the progenitor and endocrine compartments during pancreas development. *Cell Rep.* **27**, 2281-2291.e5. 10.1016/j.celrep.2019.04.083 (2019). 31116975 10.1016/j.celrep.2019.04.083 PMC6933053

[58] Singh, R. *et al.* Enhanced structure and function of human pluripotent stem cell-derived beta-cells cultured on extracellular matrix. *Stem Cells Transl. Med.* **10**, 492–505. 10.1002/sctm.20-0224 (2021). 33145960 10.1002/sctm.20-0224 PMC7900592

[59] Mamidi, A. *et al.* Mechanosignalling via integrins directs fate decisions of pancreatic progenitors. *Nature* **564**, 114–118. 10.1038/s41586-018-0762-2 (2018). 30487608 10.1038/s41586-018-0762-2

[60] Guimarães, C. F., Gasperini, L., Marques, A. P. & Reis, R. L. The stiffness of living tissues and its implications for tissue engineering. *Nat. Rev. Mater.* **5**, 351–370. 10.1038/s41578-019-0169-1 (2020). 10.1038/s41578-019-0169-1

[61] Kreuder, A.-E. *et al.* Inspired by the human placenta: A novel 3D bioprinted membrane system to create barrier models. *Sci. Rep.* **10**, 15606. 10.1038/s41598-020-72559-6 (2020). 32973223 10.1038/s41598-020-72559-6 PMC7515925

[62] Wex, C., Fröhlich, M., Brandstädter, K., Bruns, C. & Stoll, A. Experimental analysis of the mechanical behavior of the viscoelastic porcine pancreas and preliminary case study on the human pancreas. *J. Mech. Behav. Biomed. Mater.* **41**, 199–207. 10.1016/j.jmbbm.2014.10.013 (2015). 25460416 10.1016/j.jmbbm.2014.10.013

[63] Acun, A. *et al.* Liver donor age affects hepatocyte function through age-dependent changes in decellularized liver matrix. *Biomaterials* **270**, 120689. 10.1016/j.biomaterials.2021.120689 (2021). 33524812 10.1016/j.biomaterials.2021.120689 PMC7906943

[64] Linke, K. *et al.* Engineered liver-like tissue on a capillarized matrix for applied research. *Tissue Eng.* **13**, 2699–2707. 10.1089/ten.2006.0388 (2007). 17867928 10.1089/ten.2006.0388

[65] Zhang, D. *et al.* Highly efficient differentiation of human ES cells and iPS cells into mature pancreatic insulin-producing cells. *Cell Res.* **19**, 429–438. 10.1038/cr.2009.28 (2009). 19255591 10.1038/cr.2009.28

[66] Berger, C. *Influence of the Pancreatic Extracellular Matrix on Pancreatic Differentiation of Human Induced Pluripotent Stem Cells and Establishment of 3D Organ Models* (Universität Würzburg, 2023).

