# Supplementary material for: Generation of a pancreas derived hydrogel for the culture of hiPSC derived pancreatic endocrine cells
Source: Sci Rep. 2024 Sep 4;14:20653. doi: 10.1038/s41598-024-67327-9 (PMC11375036; doi:10.1038/s41598-024-67327-9)
Supplement: Supplementary file 1 — Supplementary Information. [file 41598_2024_67327_MOESM1_ESM.docx]

**Supplementary Information**

**Generation of a pancreas derived hydrogel for the culture of hiPSC derived pancreatic endocrine cells**

**Supplementary Figure 1**

**
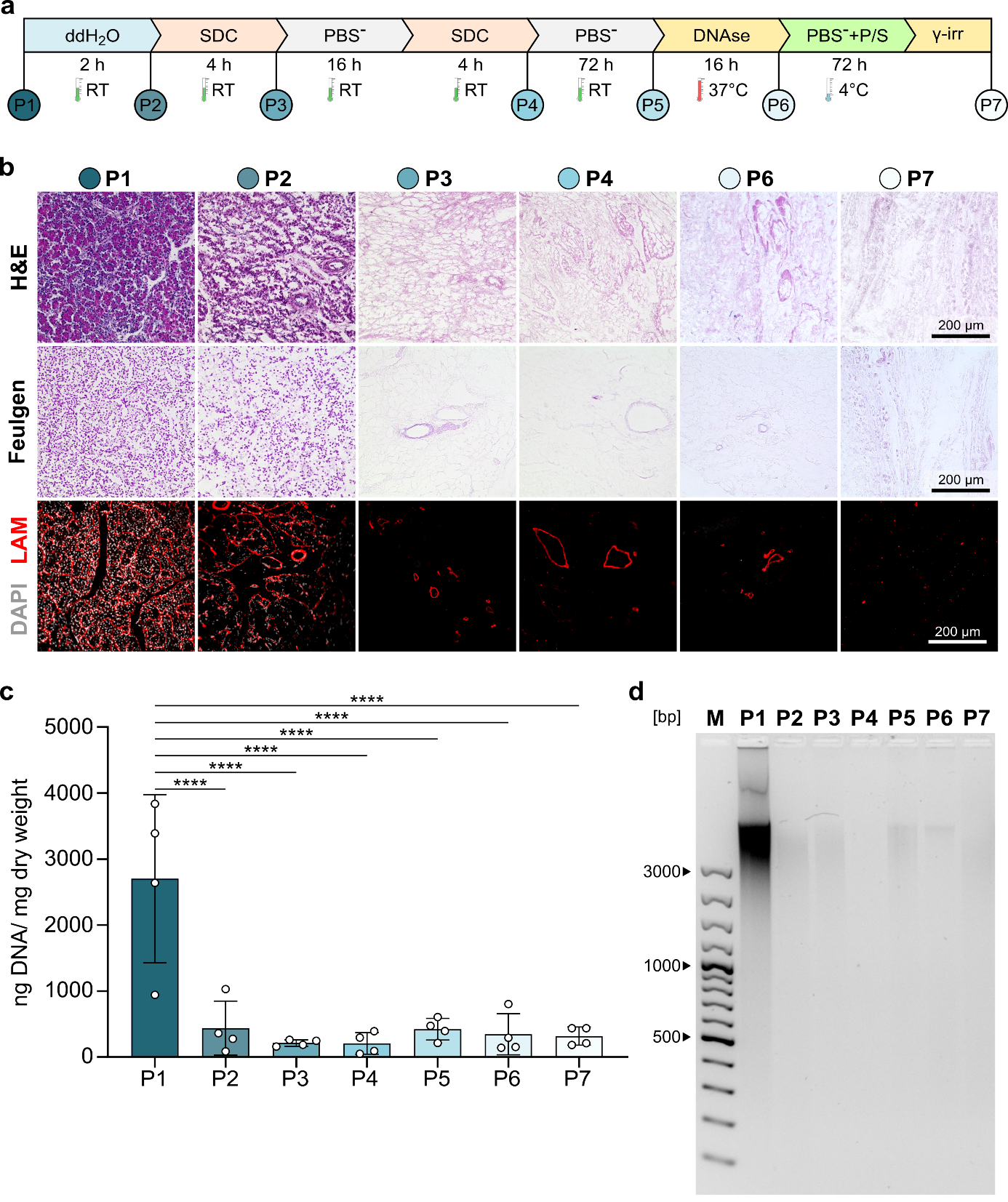
**

**Supplementary Figure 1 Step-wise deconstruction of porcine pancreas decellularization using the shortened protocol. a** Illustration of the shortened decellularization process used for the production of the PanMa. Encircled numbers correlate to the process step at which samples were taken and analyzed. **b** Tissue sections taken at different time points of decellularization and stained with Haematoxylin and Eosin (H&E) (upper panel), Feulgen (middle panel) and an antibody against laminin (red), counterstained with DAPI (grey) (lower panel) (n = 2). **c** Bar graph showing the amount of DNA per mg dry weight at different time points of pancreas decellularization. Data are shown as mean ± SD (n=4). ****P < 0.0001, one-way ANOVA with Tukey’s multiple comparisons test. **d** Samples of isolated DNA of the respective differentiation time points separated on an agarose gel. The presented images present only parts of the original gels. The uncropped images can be found in Supplementary figure 4 (n = 4).

**Supplementary Figure 2**

**
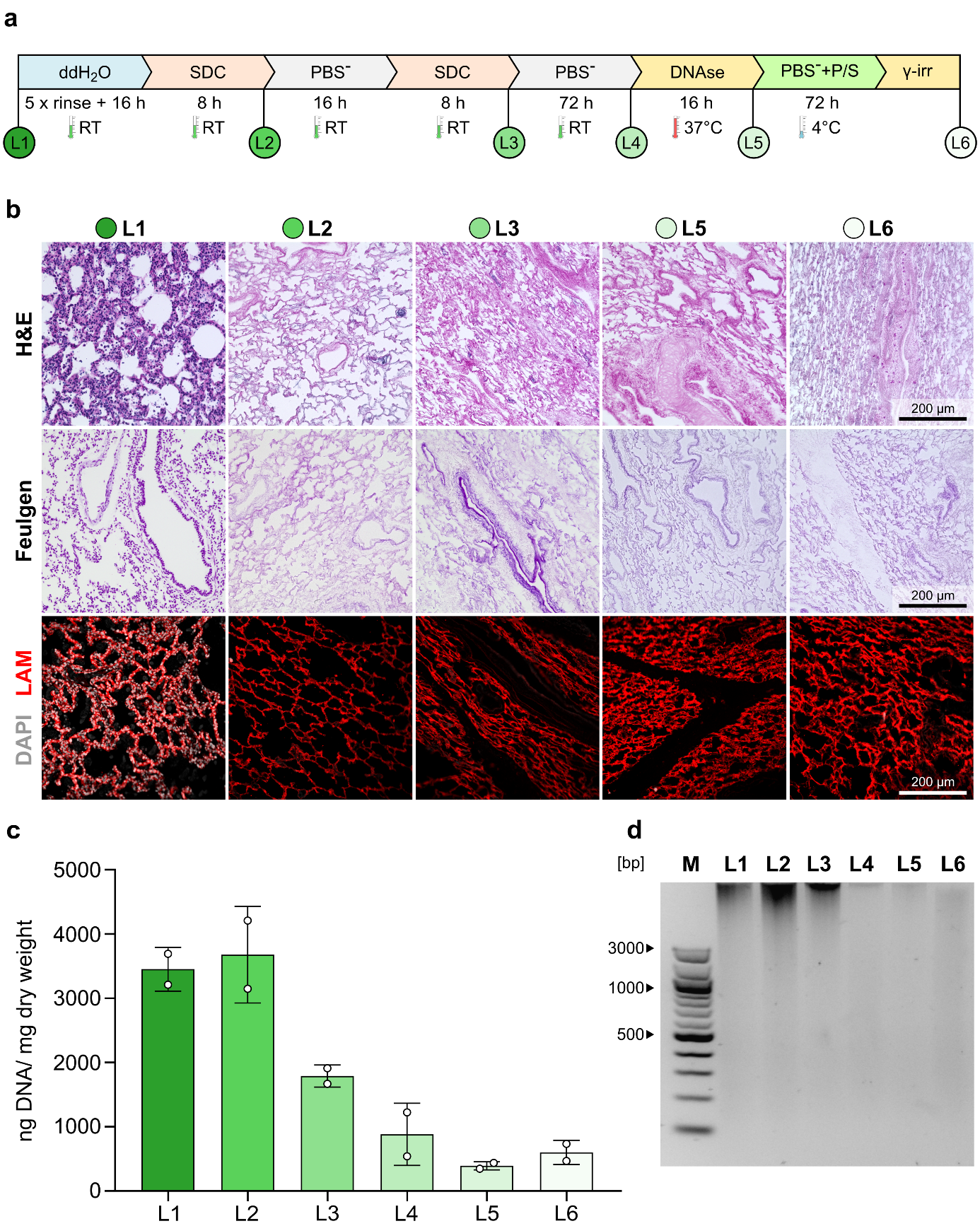
**

**Supplementary Figure 2 Step-wise deconstruction of porcine lung decellularization. a** Illustration of the decellularization process used for the production of the LungMa. Encircled numbers correlate to the process step at which samples were taken and analyzed. **b** Tissue sections taken at different time points of decellularization and stained with Haematoxylin and Eosin (H&E) (upper panel), Feulgen (middle panel) and an antibody against laminin (red), counterstained with DAPI (grey) (lower panel) (n = 2). **c** Bar graph showing the amount of DNA per mg dry weight at different time points of lung decellularization. Data are shown as mean ± SD (n=2). **d** Samples of isolated DNA of the respective differentiation time points separated on an agarose gel. The presented images present only parts of the original gels. The uncropped images can be found in Supplementary figure 4 (n = 2).

**Supplementary Figure 3**

**
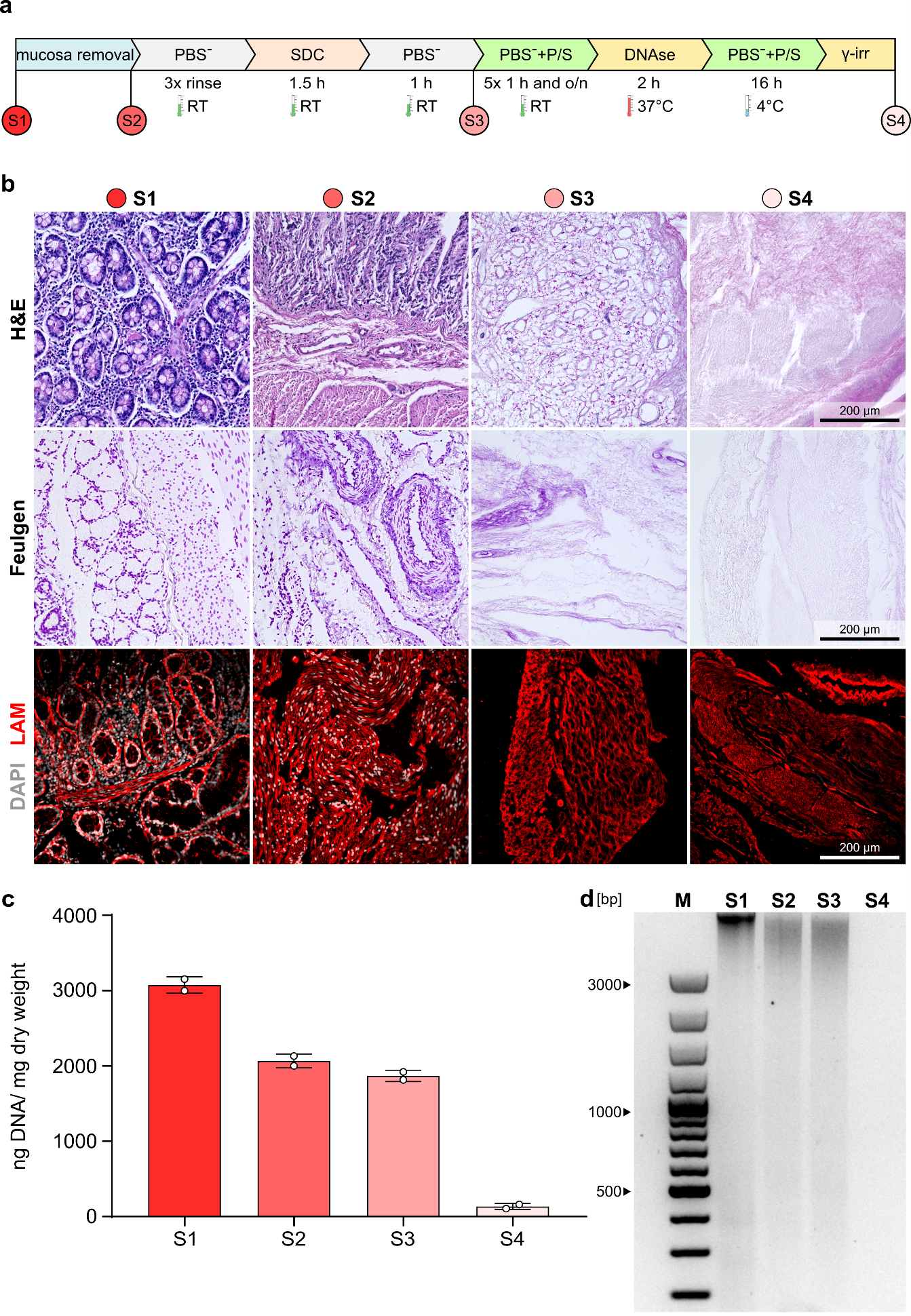
**

**Supplementary Figure 3 Step-wise deconstruction of porcine intestine decellularization. a** Illustration of the decellularization process used for the production of the SISser. Encircled numbers correlate to the process step at which samples were taken and analyzed. **b** Tissue sections taken at different time points of decellularization and stained with Haematoxylin and Eosin (H&E) (upper panel), Feulgen (middle panel) and an antibody against laminin (red), counterstained with DAPI (grey) (lower panel) (n = 2). **c** Bar graph showing the amount of DNA per mg dry weight at different time points of intestine decellularization. Data are shown as mean ± SD (n=2). **d** Samples of isolated DNA of the respective differentiation time points separated on an agarose gel. The presented images present only parts of the original gels. The uncropped images can be found in Supplementary figure 4 (n = 2).

**Supplementary Figure 4**

**
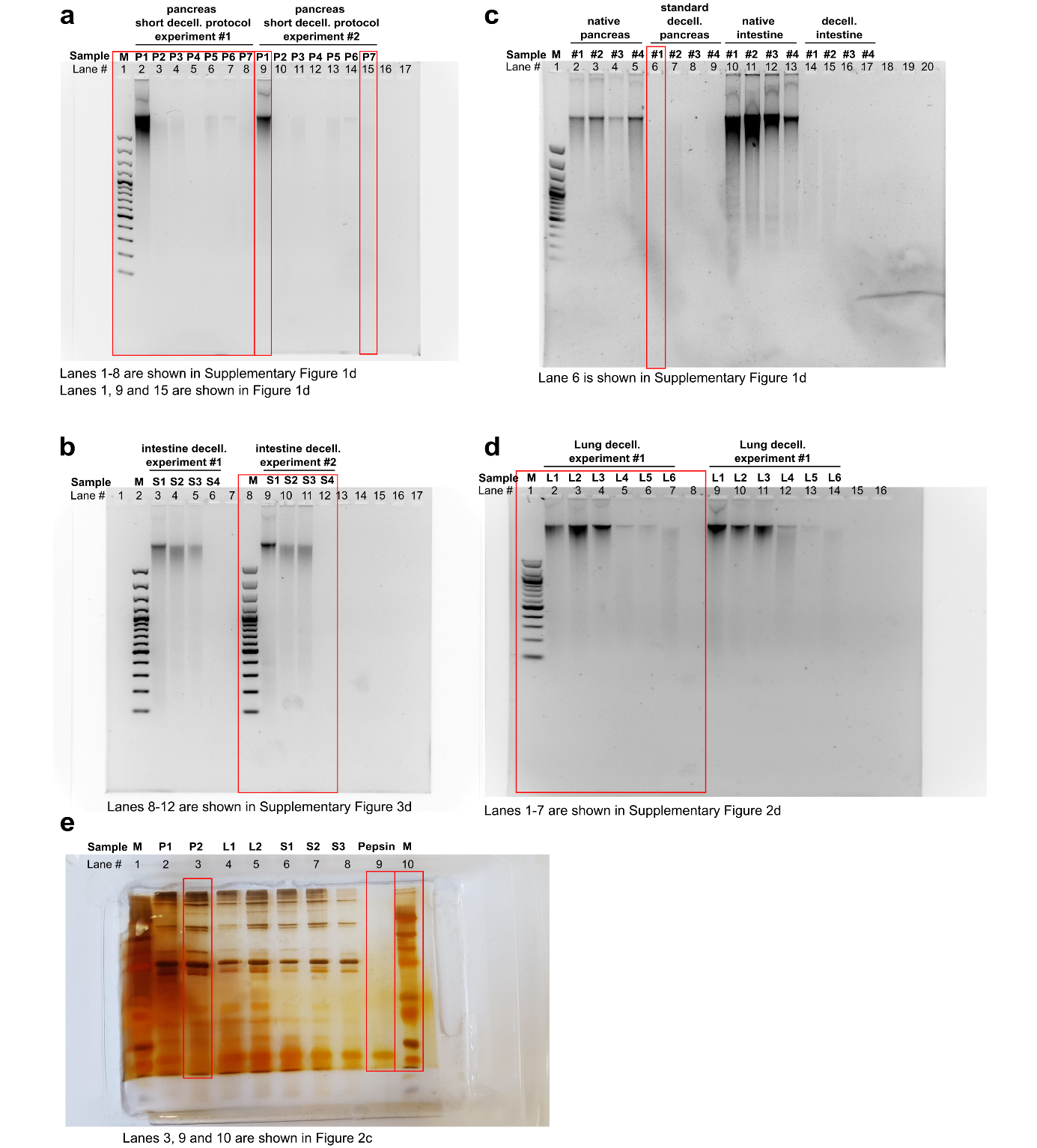
**

**Supplementary Figure 4 Full length agarose gels. a-d** Gels loaded with DNA isolated from the respective samples. Experiments #1 and #2 refers to biological replicates, whereas P1-7, L1-6 and S1-4 refer to different time points during the decellularization experiment. **e** Silver staining. P1, P2, L1, L2, S1, S2, S3 refer to different biological samples. Red boxes mark lanes that are presented in another figure of this manuscript.

**Supplementary Figure 5**


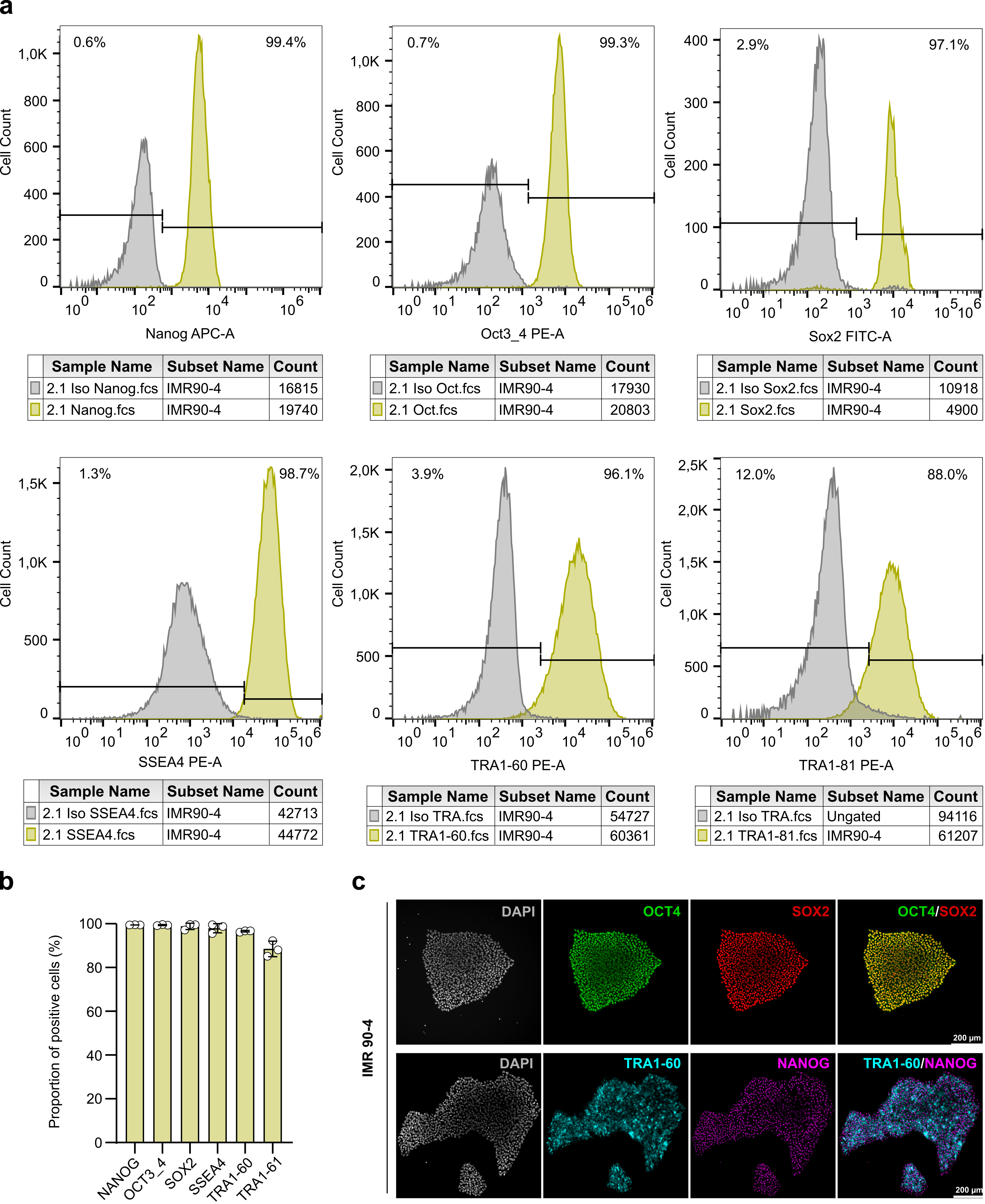


**Supplementary Figure 5 Pluripotency marker analysis of the used IMR 90-4 line. a** Representative histograms of flow cytometry experiments with IMR-90 hiPSCs immunolabeled with antibodies against NANOG, OCT3/4, SOX2, SSEA4, TRA1-60, TRA1-81 (yellow) or the respective isotype (grey). **b**Percentage of cells positive for the respective pluripotency marker of three independent experiments. **c** Representative immunofluorescence images of IMR 90-4 cells labeled with antibodies against OCT4 (green), SOX2 (red), TRA1-60 (cyan) or NANOG (magenta). Cells are counterstained with DAPI.

**Supplementary Table 1**

**Detailed information about statistical testing, sample size, compared groups and p-values.**

| **Fig. 1c** |  |  |  |  |  |  |  |  |  |  |  |  |
| --- | --- | --- | --- | --- | --- | --- | --- | --- | --- | --- | --- | --- |
| Test: one-way ANOVA, Tukey’s multiple comparisons test | | | | |  |  | P Values | <0.05 | <0.01 | <0.001 | <0.0001 |  |
| Sample size: 4 biological replicates | | | | |  |  | Scale |  |  |  |  |  |
|  |  |  |  |  |  |  | Asterisk | * | ** | *** | **** |  |
|  | native | PanMa std | PanMa short |  |  |  | Not tested | n.t. |  |  |  |  |
| native |  | 0.0023 | 0.0036 |  |  |  |  |  |  |  |  |  |
| PanMa std |  |  | 0.9508 |  |  |  |  |  |  |  |  |  |
| PanMa short |  |  |  |  |  |  |  |  |  |  |  |  |
|  |  |  |  |  |  |  |  |  |  |  |  |  |
|  |  |  |  |  |  |  |  |  |  |  |  |  |
| **Fig. 3d** |  |  |  |  |  |  |  |  |  |  |  |  |
| Test: one-way ANOVA, Sidak’s multiple comparisons test | | | | |  |  |  |  |  |  |  |  |
| Sample size: n pictures of 3 biological replicates | | | | |  |  |  |  |  |  |  |  |
|  |  |  |  |  |  |  |  |  |  |  |  |  |
| n |  | CTRL d7 | Matrigel d7 | PanMa d7 | CTRL d21 | Matrigel d21 | PanMa d21 |  |  |  |  |  |
| 10 | CTRL d7 |  | 0.9974 | 0.9573 | 0.4844 | n.t. | n.t. |  |  |  |  |  |
| 5 | Matrigel d7 |  |  | >0.9999 | n.t. | 0.4206 | n.t. |  |  |  |  |  |
| 8 | PanMa d7 |  |  |  | n.t. | n.t. | 0.3647 |  |  |  |  |  |
| 9 | CTRL d21 |  |  |  |  | 0.8403 | 0.8543 |  |  |  |  |  |
| 8 | Matrigel d21 |  |  |  |  |  | >0.9999 |  |  |  |  |  |
| 12 | PanMa d21 |  |  |  |  |  |  |  |  |  |  |  |
|  |  |  |  |  |  |  |  |  |  |  |  |  |
|  |  |  |  |  |  |  |  |  |  |  |  |  |
| **Fig. 4a** |  |  |  |  |  |  | **Fig. 4b** |  |  |  |  |  |
| Test: one-way ANOVA, Tukey’s multiple comparisons test | | | | |  |  | Test: one-way ANOVA, Tukey’s multiple comparisons test | | | | |  |
| Sample size: 3 biological replicates | | | | |  |  | Sample size: 3 biological replicates | | | | |  |
|  |  |  |  |  |  |  |  |  |  |  |  |  |
| *PDX1* |  |  |  |  |  |  | *PDX1* |  |  |  |  |  |
|  | CTRL d0 | CTRL d7 | PHydro d7 | CTRL d21 | PHydro d21 |  |  | CTRL d0 | CTRL d7 | MGel d7 | CTRL d21 | MGel d21 |
| CTRL d0 |  | 0.9942 | 0.9995 | 0.9366 | 0.9589 |  | CTRL d0 |  | 0.9136 | 0.9823 | 0.9950 | 0.9592 |
| CTRL d7 |  |  | 0.9997 | 0.9954 | 0.8224 |  | CTRL d7 |  |  | 0.9977 | 0.9895 | 0.5833 |
| PHydro d7 |  |  |  | 0.9798 | 0.8976 |  | MGel d7 |  |  |  | 0.9999 | 0.7540 |
| CTRL d21 |  |  |  |  | 0.6251 |  | CTRL d21 |  |  |  |  | 0.8296 |
| PHydro d21 |  |  |  |  |  |  | MGel d21 |  |  |  |  |  |
|  |  |  |  |  |  |  |  |  |  |  |  |  |
| *NKX6.1* |  |  |  |  |  |  | *NKX6.1* |  |  |  |  |  |
|  | CTRL d0 | CTRL d7 | PHydro d7 | CTRL d21 | PHydro d21 |  |  | CTRL d0 | CTRL d7 | MGel d7 | CTRL d21 | MGel d21 |
| CTRL d0 |  | 0.1542 | 0.5632 | 0.1638 | 0.6793 |  | CTRL d0 |  | 0.6648 | 0.8638 | 0.7820 | 0.9989 |
| CTRL d7 |  |  | 0.8434 | >0.9999 | 0.7410 |  | CTRL d7 |  |  | 0.9942 | 0.9994 | 0.5222 |
| PHydro d7 |  |  |  | 0.8613 | 0.9995 |  | MGel d7 |  |  |  | 0.9998 | 0.7404 |
| CTRL d21 |  |  |  |  | 0.7629 |  | CTRL d21 |  |  |  |  | 0.6434 |
| PHydro d21 |  |  |  |  |  |  | MGel d21 |  |  |  |  |  |
|  |  |  |  |  |  |  |  |  |  |  |  |  |
| *MAFA* |  |  |  |  |  |  | *MAFA* |  |  |  |  |  |
|  | CTRL d0 | CTRL d7 | PHydro d7 | CTRL d21 | PHydro d21 |  |  | CTRL d0 | CTRL d7 | MGel d7 | CTRL d21 | MGel d21 |
| CTRL d0 |  | 0.8298 | 0.6147 | 0.4561 | 0.8064 |  | CTRL d0 |  | 0.7803 | 0.9968 | 0.5212 | 0.9997 |
| CTRL d7 |  |  | 0.9936 | 0.9956 | >0.9999 |  | CTRL d7 |  |  | 0.9218 | 0.9886 | 0.6785 |
| PHydro d7 |  |  |  | 0.9982 | 0.9961 |  | MGel d7 |  |  |  | 0.7093 | 0.9828 |
| CTRL d21 |  |  |  |  | 0.9640 |  | CTRL d21 |  |  |  |  | 0.4227 |
| PHydro d21 |  |  |  |  |  |  | MGel d21 |  |  |  |  |  |
|  |  |  |  |  |  |  |  |  |  |  |  |  |
| *INS* |  |  |  |  |  |  | *INS* |  |  |  |  |  |
|  | CTRL d0 | CTRL d7 | PHydro d7 | CTRL d21 | PHydro d21 |  |  | CTRL d0 | CTRL d7 | MGel d7 | CTRL d21 | MGel d21 |
| CTRL d0 |  | 0.0425 | 0.2134 | 0.6726 | 0.8383 |  | CTRL d0 |  | 0.3216 | 0.9698 | 0.7671 | 0.9960 |
| CTRL d7 |  |  | 0.8180 | 0.3102 | 0.0088 |  | CTRL d7 |  |  | 0.6347 | 0.9075 | 0.1982 |
| PHydro d7 |  |  |  | 0.8571 | 0.0455 |  | MGel d7 |  |  |  | 0.9783 | 0.8635 |
| CTRL d21 |  |  |  |  | 0.2009 |  | CTRL d21 |  |  |  |  | 0.5700 |
| PHydro d21 |  |  |  |  |  |  | MGel d21 |  |  |  |  |  |
|  |  |  |  |  |  |  |  |  |  |  |  |  |
| *GCG* |  |  |  |  |  |  | *GCG* |  |  |  |  |  |
|  | CTRL d0 | CTRL d7 | PHydro d7 | CTRL d21 | PHydro d21 |  |  | CTRL d0 | CTRL d7 | MGel d7 | CTRL d21 | MGel d21 |
| CTRL d0 |  | 0.4370 | 0.7390 | 0.0524 | 0.2234 |  | CTRL d0 |  | 0.9032 | 0.9998 | 0.1609 | >0.9999 |
| CTRL d7 |  |  | 0.9794 | 0.5918 | 0.9832 |  | CTRL d7 |  |  | 0.9509 | 0.4974 | 0.9217 |
| PHydro d7 |  |  |  | 0.3261 | 0.8174 |  | MGel d7 |  |  |  | 0.2030 | >0.9999 |
| CTRL d21 |  |  |  |  | 0.8639 |  | CTRL d21 |  |  |  |  | 0.1744 |
| PHydro d21 |  |  |  |  |  |  | MGel d21 |  |  |  |  |  |
|  |  |  |  |  |  |  |  |  |  |  |  |  |
| *SST* |  |  |  |  |  |  | *SST* |  |  |  |  |  |
|  | CTRL d0 | CTRL d7 | PHydro d7 | CTRL d21 | PHydro d21 |  |  | CTRL d0 | CTRL d7 | MGel d7 | CTRL d21 | MGel d21 |
| CTRL d0 |  | 0.2127 | 0.8961 | 0.0141 | 0.0545 |  | CTRL d0 |  | 0.8706 | >0.9999 | 0.2050 | 0.9920 |
| CTRL d7 |  |  | 0.6161 | 0.4349 | 0.8882 |  | CTRL d7 |  |  | 0.9156 | 0.6386 | 0.9827 |
| PHydro d7 |  |  |  | 0.0556 | 0.2064 |  | MGel d7 |  |  |  | 0.2431 | 0.9978 |
| CTRL d21 |  |  |  |  | 0.9009 |  | CTRL d21 |  |  |  |  | 0.3626 |
| PHydro d21 |  |  |  |  |  |  | MGel d21 |  |  |  |  |  |
|  |  |  |  |  |  |  |  |  |  |  |  |  |
|  |  |  |  |  |  |  |  |  |  |  |  |  |
| **Fig. 5d+f** |  |  |  |  |  |  |  |  |  |  |  |  |
| Test: one-way ANOVA, Sidak’s multiple comparisons test | | | | |  |  |  |  |  |  |  |  |
| Sample size: n spherods of N biological replicates | | | | |  |  |  |  |  |  |  |  |
|  |  |  |  |  |  |  |  |  |  |  |  |  |
|  |  | PDX1 nucl. |  |  |  |  |  |  |  |  |  |  |
| N | n |  | CTRL d0 | CTRL d7 | PHydro d7 | MGel d7 | CTRL d21 | PHydro d21 | MGel d21 |  |  |  |
| 4 | 55 | CTRL d0 |  | <0.0001 | n.t. | n.t. | <0.0001 | n.t. | n.t. |  |  |  |
| 6 | 79 | CTRL d7 |  |  | 0.9986 | >0.9999 | 0.1649 | n.t. | n.t. |  |  |  |
| 3 | 22 | PHydro d7 |  |  |  | 0.9802 | n.t. | >0.9999 | n.t. |  |  |  |
| 3 | 22 | MGel d7 |  |  |  |  | n.t. | n.t. | >0.9999 |  |  |  |
| 4 | 32 | CTRL d21 |  |  |  |  |  | 0.3943 | 0.7684 |  |  |  |
| 3 | 21 | PHydro d21 |  |  |  |  |  |  | 0.9997 |  |  |  |
| 4 | 32 | MGel d21 |  |  |  |  |  |  |  |  |  |  |
|  |  |  |  |  |  |  |  |  |  |  |  |  |
|  |  | NKX6.1 nucl. |  |  |  |  |  |  |  |  |  |  |
|  |  |  | CTRL d0 | CTRL d7 | PHydro d7 | MGel d7 | CTRL d21 | PHydro d21 | MGel d21 |  |  |  |
| 4 | 52 | CTRL d0 |  | <0.0001 | n.t. | n.t. | 0.6730 | n.t. | n.t. |  |  |  |
| 6 | 79 | CTRL d7 |  |  | >0.9999 | 0.9979 | 0.3413 | n.t. | n.t. |  |  |  |
| 3 | 21 | PHydro d7 |  |  |  | 0.9914 | n.t. | 0.0159 | n.t. |  |  |  |
| 3 | 20 | MGel d7 |  |  |  |  | n.t. | n.t. | 0.0005 |  |  |  |
| 4 | 32 | CTRL d21 |  |  |  |  |  | 0.7298 | 0.0038 |  |  |  |
| 3 | 20 | PHydro d21 |  |  |  |  |  |  | 0.7536 |  |  |  |
| 4 | 28 | MGel d21 |  |  |  |  |  |  |  |  |  |  |
|  |  |  |  |  |  |  |  |  |  |  |  |  |
|  |  | MAFA nucl. |  |  |  |  |  |  |  |  |  |  |
|  |  |  | CTRL d0 | CTRL d7 | PHydro d7 | MGel d7 | CTRL d21 | PHydro d21 | MGel d21 |  |  |  |
| 4 | 58 | CTRL d0 |  | <0.0001 | n.t. | n.t. | <0.0001 | n.t. | n.t. |  |  |  |
| 6 | 80 | CTRL d7 |  |  | 0.0428 | 0.0179 | 0.2332 | n.t. | n.t. |  |  |  |
| 3 | 19 | PHydro d7 |  |  |  | >0.9999 | n.t. | 0.1301 | n.t. |  |  |  |
| 3 | 24 | MGel d7 |  |  |  |  | n.t. | n.t. | 0.0002 |  |  |  |
| 4 | 31 | CTRL d21 |  |  |  |  |  | 0.0044 | 0.0033 |  |  |  |
| 3 | 15 | PHydro d21 |  |  |  |  |  |  | >0.0001 |  |  |  |
| 4 | 30 | MGel d21 |  |  |  |  |  |  |  |  |  |  |
|  |  |  |  |  |  |  |  |  |  |  |  |  |
|  |  | CPEP area |  |  |  |  |  |  |  |  |  |  |
|  |  |  | CTRL d0 | CTRL d7 | PHydro d7 | MGel d7 | CTRL d21 | PHydro d21 | MGel d21 |  |  |  |
| 4 | 54 | CTRL d0 |  | 0.9662 | n.t. | n.t. | 0.0018 | n.t. | n.t. |  |  |  |
| 6 | 78 | CTRL d7 |  |  | 0.8630 | 0.0002 | <0.0001 | n.t. | n.t. |  |  |  |
| 3 | 19 | PHydro d7 |  |  |  | 0.1313 | n.t. | 0.1465 | n.t. |  |  |  |
| 3 | 15 | MGel d7 |  |  |  |  | n.t. | n.t. | <0.0001 |  |  |  |
| 4 | 32 | CTRL d21 |  |  |  |  |  | 0.2198 | 0.9558 |  |  |  |
| 3 | 22 | PHydro d21 |  |  |  |  |  |  | 0.9791 |  |  |  |
| 4 | 22 | MGel d21 |  |  |  |  |  |  |  |  |  |  |
|  |  |  |  |  |  |  |  |  |  |  |  |  |
|  |  | GCG area |  |  |  |  |  |  |  |  |  |  |
|  |  |  | CTRL d0 | CTRL d7 | PHydro d7 | MGel d7 | CTRL d21 | PHydro d21 | MGel d21 |  |  |  |
| 4 | 54 | CTRL d0 |  | 0.0061 | n.t. | n.t. | 0.0001 | n.t. | n.t. |  |  |  |
| 6 | 78 | CTRL d7 |  |  | 0.7670 | 0.7704 | 0.5561 | n.t. | n.t. |  |  |  |
| 3 | 19 | PHydro d7 |  |  |  | 0.1738 | n.t. | <0.0001 | n.t. |  |  |  |
| 3 | 15 | MGel d7 |  |  |  |  | n.t. | n.t. | 0.0117 |  |  |  |
| 4 | 32 | CTRL d21 |  |  |  |  |  | 0.1938 | 0.0020 |  |  |  |
| 3 | 22 | PHydro d21 |  |  |  |  |  |  | <0.0001 |  |  |  |
| 4 | 22 | MGel d21 |  |  |  |  |  |  |  |  |  |  |
|  |  |  |  |  |  |  |  |  |  |  |  |  |
|  |  | SST area |  |  |  |  |  |  |  |  |  |  |
|  |  |  | CTRL d0 | CTRL d7 | PHydro d7 | MGel d7 | CTRL d21 | PHydro d21 | MGel d21 |  |  |  |
| 4 | 54 | CTRL d0 |  | >0.9999 | n.t. | n.t. | 0.2132 | n.t. | n.t. |  |  |  |
| 6 | 78 | CTRL d7 |  |  | 0.0142 | 0.1026 | 0.1241 | n.t. | n.t. |  |  |  |
| 3 | 19 | PHydro d7 |  |  |  | 0.0001 | n.t. | 0.0021 | n.t. |  |  |  |
| 3 | 15 | MGel d7 |  |  |  |  | n.t. | n.t. | 0.4883 |  |  |  |
| 4 | 32 | CTRL d21 |  |  |  |  |  | 0.0172 | 0.2604 |  |  |  |
| 3 | 22 | PHydro d21 |  |  |  |  |  |  | 0.9959 |  |  |  |
| 4 | 22 | MGel d21 |  |  |  |  |  |  |  |  |  |  |
|  |  |  |  |  |  |  |  |  |  |  |  |  |
|  |  |  |  |  |  |  |  |  |  |  |  |  |
| **Fig. S1c** |  |  |  |  |  |  |  |  |  |  |  |  |
| Test: one-way ANOVA, Tukey’s multiple comparisons test | | | | |  |  |  |  |  |  |  |  |
| Sample size: 4 biological replicates | | | | |  |  |  |  |  |  |  |  |
|  |  |  |  |  |  |  |  |  |  |  |  |  |
|  | P1 | P2 | P3 | P4 | P5 | P6 | P7 |  |  |  |  |  |
| P1 |  | <0.0001 | <0.0001 | <0.0001 | <0.0001 | <0.0001 | <0.0001 |  |  |  |  |  |
| P2 |  |  | n.t. | n.t. | n.t. | n.t. | n.t. |  |  |  |  |  |
| P3 |  |  |  | n.t. | n.t. | n.t. | n.t. |  |  |  |  |  |
| P4 |  |  |  |  | n.t. | n.t. | n.t. |  |  |  |  |  |
| P5 |  |  |  |  |  | n.t. | n.t. |  |  |  |  |  |
| P6 |  |  |  |  |  |  | n.t. |  |  |  |  |  |
| P7 |  |  |  |  |  |  |  |  |  |  |  |  |
|  |  |  |  |  |  |  |  |  |  |  |  |  |
|  |  |  |  |  |  |  |  |  |  |  |  |  |
| **Fig. S2c** |  |  |  |  |  |  |  |  |  |  |  |  |
| Test: None due to low sample size | | | | |  |  |  |  |  |  |  |  |
| Sample size: 2 biological replicates | | | | |  |  |  |  |  |  |  |  |
|  |  |  |  |  |  |  |  |  |  |  |  |  |
|  |  |  |  |  |  |  |  |  |  |  |  |  |
| **Fig. S3c** |  |  |  |  |  |  |  |  |  |  |  |  |
| Test: None due to low sample size | | | | |  |  |  |  |  |  |  |  |
| Sample size: 2 biological replicates | | | | |  |  |  |  |  |  |  |  |

**Supplementary Table 2**

**Code for image J Macros.**

| **Name** | **Code** |
| --- | --- |
| **Macro #1** | //DAPI count (PDX1)  run("Set Scale...", "distance=0 known=0 unit=pixel");  run("8-bit");  run("Subtract Background...", "rolling=25 sliding");  run("Enhance Contrast...", "saturated=0.01 normalize");  run("Mean...", "radius=3");  run("Auto Threshold", "method=Huang");  run("Adjustable Watershed", "tolerance=0.3");  run("Analyze Particles...", "size=60-Infinity show=Outlines exclude include summarize"); |
| **Macro #2** | //PDX1 count  run("Set Scale...", "distance=0 known=0 unit=pixel");  run("8-bit");  run("Enhance Contrast...", "saturated=0.001 normalize");  run("Subtract Background...", "rolling=5 sliding");  run("Mean...", "radius=2");  run("Auto Threshold", "method=Otsu");  run("Adjustable Watershed", "tolerance=0.3");  run("Analyze Particles...", "size=60-Infinity circularity=0.60-1.00 show=Outlines exclude include summarize"); |
| **Macro #3** | //DAPI count (NKX6.1)  run("Set Scale...", "distance=0 known=0 unit=pixel");  run("8-bit");  run("Subtract Background...", "rolling=25 sliding");  run("Enhance Contrast...", "saturated=0.01 normalize");  run("Mean...", "radius=3");  run("Auto Threshold", "method=Huang");  run("Adjustable Watershed", "tolerance=0.3");  run("Analyze Particles...", "size=20-Infinity show=Outlines exclude include summarize"); |
| **Macro #4** | //NKX6.1 count  run("Set Scale...", "distance=0 known=0 unit=pixel");  run("8-bit");  run("Enhance Contrast...", "saturated=0.05 normalize");  run("Subtract Background...", "rolling=20 sliding");  run("Mean...", "radius=2");  run("Auto Threshold", "method=Otsu");  run("Adjustable Watershed", "tolerance=0.3");  run("Analyze Particles...", "size=20-Infinity circularity=0.60-1.00 show=Outlines exclude include summarize"); |
| **Macro #5** | //DAPI count (MAFA)  run("Set Scale...", "distance=0 known=0 unit=pixel");  run("8-bit");  run("Subtract Background...", "rolling=25 sliding");  run("Enhance Contrast...", "saturated=0.01 normalize");  run("Mean...", "radius=3");  run("Auto Threshold", "method=Huang");  run("Adjustable Watershed", "tolerance=0.3");  run("Analyze Particles...", "size=60-Infinity show=Outlines exclude include summarize"); |
| **Macro #6** | //MAFA count  run("Set Scale...", "distance=0 known=0 unit=pixel");  run("8-bit");  run("Subtract Background...", "rolling=5 sliding");  run("Mean...", "radius=2");  run("Auto Threshold", "method=Moments");  run("Adjustable Watershed", "tolerance=0.5");  run("Analyze Particles...", "size=60-Infinity circularity=0.60-1.00 show=Outlines exclude include summarize"); |
| **Macro #7** | //DAPI Area  run("Set Scale...", "distance=0 known=0 pixel=1 unit=pixel");  run("8-bit");  run("Subtract Background...", "rolling=50 sliding");  run("Mean...", "radius=1");  run("Auto Threshold", "method=Otsu white");  run("Analyze Particles...", "size=0-Infinity show=Outlines display clear summarize add"); |
| **Macro #8** | //CPEP Area  run("Set Scale...", "distance=0 known=0 pixel=1 unit=pixel");  run("8-bit");  run("Subtract Background...", "rolling=50 sliding");  run("Mean...", "radius=1");  run("Auto Threshold", "method=IsoData white");  run("Analyze Particles...", "size=0-Infinity show=Outlines display clear summarize add"); |
| **Macro #9** | //GCG Area  run("Set Scale...", "distance=0 known=0 pixel=1 unit=pixel");  run("8-bit");  run("Subtract Background...", "rolling=50 sliding");  run("Mean...", "radius=1");  run("Auto Threshold", "method=IsoData white")  run("Analyze Particles...", "size=0-Infinity show=Outlines display clear summarize add"); |
| **Macro #10** | //SST Area  run("Set Scale...", "distance=0 known=0 pixel=1 unit=pixel");  run("8-bit");  run("Subtract Background...", "rolling=50 sliding");  run("Mean...", "radius=1");  run("Auto Threshold", "method=IsoData white");  run("Analyze Particles...", "size=0-Infinity show=Outlines display clear summarize add"); |
| **Macro #11** | //FDA area  run("Subtract Background...", "rolling=400 sliding");  run("Set Scale...", "distance=0 known=0 unit=pixel");  run("Mean...", "radius=2");  run("8-bit");  run("Auto Threshold", "method=Triangle white");  run("Analyze Particles...", " show=Outlines display clear summarize add"); |
| **Macro #12** | //PI area  run("Subtract Background...", "rolling=100 sliding");  run("Set Scale...", "distance=0 known=0 unit=pixel");  run("Mean...", "radius=2");  run("8-bit");  run("Auto Threshold", "method=Triangle white");  run("Analyze Particles...", " show=Outlines display clear summarize add"); |
